# Innovative hydrogels in cutaneous wound healing: current status and future perspectives

**DOI:** 10.3389/fbioe.2025.1454903

**Published:** 2025-05-12

**Authors:** Prasad Sawadkar, Ferdinand Lali, Elena Garcia-Gareta, Beatriz Gil Garrido, Abdullah Chaudhry, Priya Matharu, Christos Kyriakidis, Karin Greco

**Affiliations:** ^1^ The Griffin Institute - Northwick Park Institute for Medical Research, Northwick Park and St Mark’s Hospitals, London, United Kingdom; ^2^ Division of Surgery and Interventional Science, University College London, London, United Kingdom; ^3^ Regenerative Biomaterials Group, The RAFT Institute and The Griffin Institute, Northwick Park and Saint Mark’s Hospital, London, United Kingdom; ^4^ Division of Biomaterials and Tissue Engineering, Eastman Dental Institute, University College London, London, United Kingdom; ^5^ Multiscale in Mechanical and Biological Engineering Research Group, School of Engineering and Architecture, Aragon Institute of Engineering Research, University of Zaragoza, Zaragoza, Spain; ^6^ Aragon Institute for Health Research (IIS Aragon), Miguel Servet University Hospital, Zaragoza, Aragon, Spain

**Keywords:** injectable hydrogels, wound healing, polymers, extracellular matrix, biomaterials, chronic wounds

## Abstract

Chronic wounds pose a substantial burden on healthcare systems, necessitating innovative tissue engineering strategies to enhance clinical outcomes. Hydrogels, both of natural and synthetic origin, have emerged as versatile biomaterials for wound management due to their structural adaptability, biocompatibility, and tunable physicochemical properties. Their hydrophilic nature enables efficient nutrient transport, waste removal, and cellular integration, while their malleability facilitates application to deep and irregular wounds, providing an optimal microenvironment for cell adhesion, proliferation, and differentiation. Extracellular matrix (ECM)- based hydrogels retain bioactive molecules that support cellular infiltration, immune modulation, and tissue remodelling, making them highly effective scaffolds for growth factor delivery and regenerative therapies. Additionally, their injectability and potential for *in situ* polymerization enable minimally invasive applications, allowing on-demand gelation at target sites. By modifying their mechanical properties through crosslinking, hydrogels can achieve enhanced structural stability, prolonged degradation control, and improved surgical handling, optimizing their functionality in dynamic wound environments. This review outlines current approaches to skin tissue engineering, examining the biomaterials employed in hydrogel design, their limitations, and their interactions with host tissues. Furthermore, it highlights the emerging potential of functionalized injectable hydrogels, particularly those engineered for controlled drug release, enhanced bioactivity, and patient-specific therapeutic applications. These hydrogels offer a transformative platform for advanced wound care and regenerative medicine.

## 1 Introduction

Chronic wounds are generally defined as persistent full thickness or superficial skin loss that for different reasons fail to heal within the normal timeframe (Falanga et al., 2022). They are often the result of underlying conditions like diabetes, which is tightly associated with chronic wounds such as diabetic foot ulcers (DFU) or venous leg ulcers (VLU). The worldwide estimated prevalence of chronic wounds ranges between 1.47 and 2.2 per 1,000 population (Martinengo et al., 2019). In the UK, a cohort study gathering 2017/2018 data showed an estimated 3.8 million skin wound patients treated by the NHS, out of which more than 1.5 million suffered from chronic wounds including DFU, VLU and pressure ulcers (PU) (Guest et al., 2020).

The clinical management of chronic wounds requires the removal of non-viable wound components from the wound bed in a process known as debridement and infection control. Once the wound is clean, the wound bed is covered by a dressing for protection, moisture (exudate) management and compression to facilitate the healing process (Eriksson et al., 2022).

Traditional wound dressings aim to physically isolate and compress the wound; nevertheless, advances in tissue engineering have allowed the development of multifunctional dressings using biomaterials.

**FIGURE 1:**
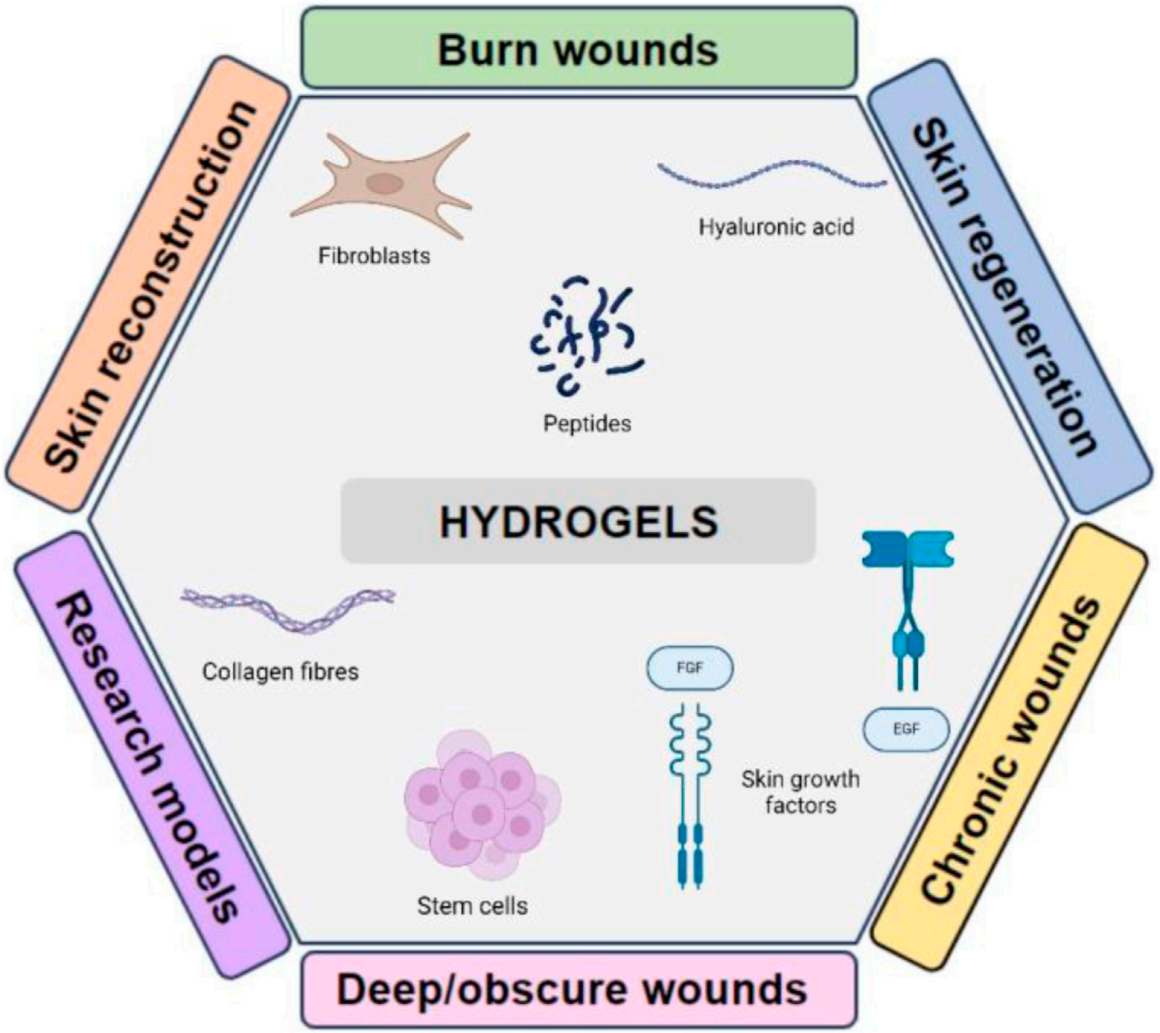
Diagram showing an overview of the predominant applications of hydrogels and the possible biomolecules that can be incorporated to alter their characteristics.

Biomaterials have significantly impacted medical interventions, leading to significant progress in areas such as tissue engineering, drug discovery, delivery of small molecules, implants, immunotherapies, and wound healing (Figure 1) (Li and Mooney, 2016; Dimatteo et al., 2018; Zhang X. et al., 2020; Lupu et al., 2023). Their immense potential stems from their capacity to offer physical support and to serve as carriers for biologically active substances such as cells, growth factors, biomolecules, and drugs. Furthermore, they can be chemically and physio-chemically modified to engineer specific functionalities (Kim et al., 2019; La Manna et al., 2021) Biomaterials encompass a diverse array of compounds with varying functions and structural characteristics, spanning from naturally occurring biological molecules to synthetic polymers (Kim et al., 2019).

The selection of a suitable scaffold composition relies on biomaterials biocompatibility and critical functionality for specific purposes. In wound healing, one key factor to consider when designing a scaffold capacity to facilitate the infiltration and proper differentiation of the crucial cell types in the skin, which ultimately restores both form and function. Ideally, biological scaffolds should possess sufficient stability to allow for tissue incorporation and subsequent remodelling. One way to accomplish this is by utilising exogenous crosslinking agents. Depending on the chosen cross-linking agent, variations in production chemistry can alter the physical features of scaffolds (Kennedy et al., 2011).

The long-term remodelling outcome varies significantly across different biomaterials and their physicochemical properties (Halim et al., 2010). Studies conducted by our research group have shown that non-crosslinked biomaterials exhibit superior biocompatibility compared to crosslinked biomaterials (Kennedy et al., 2011; Azagarsamy and Anseth, 2013). However, the usage of crosslinked scaffolds may be beneficial in specific applications to decrease the rate of degradation, primarily if the scaffold is meant to be a permanent and non-absorbable matrix. The chronological order of host-initiated remodelling activities can be used to predict the outcome of wound healing (Kennedy et al., 2011; Van Vlierberghe et al., 2011).

Most of these novel dressings are available in the form of sheets, which can be either single-layered for repairing the epidermis or dermis; or bilayered, for replacing both the epidermis and dermis (Kim and Jacobs, 1996; Ifkovits and Burdick, 2007; Scheideler et al., 2007; Azagarsamy and Anseth, 2013; Alina et al., 2020; Pineda-Castañeda et al., 2023). They can be seeded with appropriate cells, such as dermal fibroblasts or epidermal keratinocytes to stimulate would healing, or they can be completely devoid of cells to act as a scaffold for new tissue deposition (Shi et al., 2006). Although successful, skin replacements pose several challenges such as delayed engraftment and unsatisfactory wound healing results, rejection, and difficulty in handling.

Skin replacement approaches have also been taken for the management of non-healing wounds, like burns. Surgical procedures for non-healing wounds often use autografts, allografts, or xenografts. In this approach, split-thickness dermo-epithelial donor skin is utilised to provide coverage for the wound’s surface and the damaged dermis. Autografts are the preferable choice in order to circumvent the issues of infection and rejection that are commonly linked with the utilisation of allografts or xenografts. Nevertheless, the utilisation of autografts is constrained by the accessibility of unblemished skin on the burn victim and the adverse effects on the donor site after harvesting (Park, 1999). Other challenges in the use of autografts or other forms of skin substitutes in burn victims include the fragility of epidermal sheets in cutaneous wound grafting and the flexibility of materials required to fill irregular wound shapes. Injuries that affect numerous layers of the skin can cause intense pain and anguish and present challenges in terms of treatment options.

One solution that has gained recognition over the last few decades, both from a clinical and academic point-of-view, is hydrogel-based skin substitutes. Hydrogels are three-dimensional (3D) hydrophilic polymer networks that can absorb up to 99% of their weight in water. They present clear advantages for wound healing applications, like moisture control, non-damaging adhesion and biocompatibility, besides providing physical isolation of the wound bed. In addition, the internal structure of hydrogels makes them semi-permeable to gases, allowing oxygenation of the wound bed (Tavakoli and Klar, 2020). Hydrogels can easily be tailored to modify their mechanical and physical properties to mimic native skin structures, providing a reliable scaffold for tissue remodelling and speeding the wound healing process. The aqueous nature and polymeric structure of hydrogels allow for the addition of bioactive components. Besides cells, which had already been incorporated in other forms of skin replacement, hydrogels can hold antibacterial and therapeutic agents, both in the form of nanoparticles (Dam et al., 2023) or intrinsic in the hydrogel formulation to enhance full healing (Gao et al., 2019; Tatarusanu et al., 2023) Attempts have been made to create hydrogels in the form of sheets, powders, and slurry to ensure full wound covering andstimulate the regenerative response of the host tissue (Park, 1999; Shi et al., 2006; Garbern et al., 2011; Chen et al., 2017; Miranda-Calderon et al., 2022).

Skin wounds frequently exhibit uneven contours that pose challenges in achieving complete coverage with sheet-like skin substitutes (Chen et al., 2017). Hydrogels can be administered in a gel form or allowed to solidify in the body, reducing the need for surgery. Alternative delivery methods for *in situ* gelable hydrogels are being developed to improve adaptability to wound bed shape for long term management. Efforts are particularly strong on the development of spray and injectable hydrogels (Zhang et al., 2011; Wu et al., 2022; Nejati and Mongeau, 2023; Nishiguchi, 2024). Injectable hydrogels are particularly promising because of the high efficacy of local administration to target site (Nishiguchi, 2024).

This review explores the functional and mechanical advances of hydrogel technology to support the wound healing process. Particularly, it outlines the current commercially available options for skin substitutes and their limitations and focuses on the potential of hydrogel technologies to address them. In addition, it reviews the current challenges in the clinical use of hydrogels and state-of-art with a focus on injectable hydrogels as promising solutions.

## 2 Wound healing and wound types

Wound healing is an intricate and tightly controlled process that plays a crucial role in preserving the protective function of the skin (Zhang X. et al., 2020). The healing process includes the sequential stages of haemostasis, inflammation, proliferation, and remodelling, which require precise interactions between different cell types, cytokines, growth factors, proteases, and ECM components to facilitate the creation of new tissue and the closure of wounds (Lupu et al., 2023).

Following an injury, the first phase of healing drives vasoconstriction ensues to minimise blood loss in a process known as haemostasis. Platelets attach to the exposed extracellular matrix (ECM) at the injury site, creating a provisional fibrin clot. This clot functions as a temporary barrier and a reservoir for growth factors, including platelet-derived growth factor (PDGF) and transforming growth factor-beta (TGF-β), which activate the healing cascade.

The inflammatory phase is marked by the invasion of immune cells, chiefly neutrophils and macrophages. Neutrophils are the primary responders, eliminating pathogens and cellular debris by phagocytosis. Macrophages perform a dual function in phagocytosis and the release of cytokines [e.g., interleukin-1 (IL-1), tumour necrosis factor-alpha (TNF-α)] and growth factors [e.g., TGF-β, vascular endothelial growth factor (VEGF)]. These signalling chemicals attract supplementary cells and facilitate the transition to the proliferative phase.

During the proliferative phase, the wound undergoes re-epithelialization, angiogenesis, and ECM deposition. Keratinocytes traverse from the wound peripheries to re-establish the epithelial barrier. Fibroblasts multiply and produce collagen type III, which constitutes the granulation tissue. Simultaneously, endothelial cells are activated by VEGF to generate new blood vessels, so assuring sufficient oxygen and nutrient delivery. Myofibroblasts, characterised by the expression of alpha-smooth muscle actin (α-SMA), promote wound contraction. The concluding phase entails the development and reorganisation of the extracellular matrix (ECM). Collagen type III is progressively substituted by collagen type I, enhancing tensile strength. Matrix metalloproteinases (MMPs) and tissue inhibitors of metalloproteinases (TIMPs) govern the breakdown and production of extracellular matrix (ECM) components.

Gradually, the wound evolves from hypercellular, vascular granulation tissue to a more acellular, avascular scar. Healing outcomes can either be regenerative, meaning they restore the shape and function of the injured tissue, or reparative, where the wound closes but the underlying tissue architecture and functions are not fully restored. Therefore, when wounds are significant, the healing process results in the formation of scars that may impair the skin’s functionality. Although the epidermis undergoes constant remodelling and regeneration following damage, the regeneration of lost dermal tissue is limited and typically results in scar formation (Kim et al., 2019; Alina et al., 2020; La Manna et al., 2021).

Scar tissue not only has a negative impact on appearance but also leads to contracture, which restricts movement, particularly around joints. Additional factors contributing to impaired wound healing involve the inability to develop new blood vessels to supply the wound area and the inability to properly respond to growth factors. These issues may arise due to the breakdown of growth factors by matrix Metalloproteinases (MMPs) found in the wound fluid or due to alterations in receptor expression by the cells present in the chronic wound (Halim et al., 2010; Kennedy et al., 2011; Pineda-Castañeda et al., 2023). According to reports, wounds that do not heal are resistant to wound-healing mediators like PDGF (Miranda-Calderon et al., 2022).

### 2.1 Wound types

Depending on the depth of the lesion, wounds can be classed as epidermal, superficial, deep dermal or full thickness. Although healing phases are consistent for all wounds, the specific process is contingent upon each wounds’ individual characteristics. Cutaneous wounds are categorised based on the nature of the cut and the specific layers that are impacted. These wounds can be classified as either incisional or excisional.

Incisional wounds result in minimum tissue loss and the wound edges come together naturally. Stitches may be used to stabilise the wound and promote healing through a process called primary intention. Excisional wounds heal through secondary intention, in which lost dermal tissue is replaced by granulation tissue. Granulation tissue is characterised by the presence of newly formed capillaries that originate from the borders of the wound, as well as invading fibroblasts that deposit the new ECM. A new epidermis grows on the recently deposited ECM to close the wound. This is a tightly regulated process: it has been shown that an excessive expression of fibrovascular tissue might result in scarring complications during the latter phases of wound healing whereas insufficient granulation tissue in hard-to-heal wounds hinders the process of epithelial closure and ongoing remodelling (De Castro Brás et al., 2010; Shevchenko et al., 2010; Dimatteo et al., 2018). Thus, wound healing therapies primarily aim to efficiently restore the dermal layer in order to facilitate wound closure by epithelial covering (Kennedy et al., 2011; Dimatteo et al., 2018).

The resolution of inflammation and advancing the proliferative and remodelling phases are disrupted in difficult-to-heal deep dermal or full-thickness wounds, such as those caused by burn injuries and chronic wounds like pressure sores and diabetic ulcers (Kim and Jacobs, 1996; Azagarsamy and Anseth, 2013). In those cases, the healing process is altered due to systemic and local causes. There are several factors that can negatively impact the healing in hard-to-heal wounds. Local factors encompass local tissue oxygenation and infection, while the systemic factors comprise stress, diabetes, obesity, medicines, alcoholism, smoking, nutritional status and age. During aging, the thinning of the skin and the decreased ability of older dermal fibroblasts to multiply ultimately reduces the regeneration capabilities of the aged dermis (Halim et al., 2010; Kennedy et al., 2011; Pineda-Castañeda et al., 2023).

Chronic wounds frequently have an extended period of inflammation when healing, which prevents the regeneration of skin and leads to increased scarring. In severe deep partial-thickness and full-thickness wounds, surgical intervention is necessary to repair the lost dermis (Azagarsamy and Anseth, 2013).

## 3 Traditional and commercial skin substitutes

Skin substitutes are categorised into three types: those aimed to replace the epidermis, those designed to replace the dermis, and those intended to replace both. The last type is referred to as a full-thickness skin substitute (Zhang X. et al., 2020). There are several commercially available examples of all three types, as shown in Table 1.

**TABLE 1:** Commercially available epidermal substitutes, dermal substitutes and bilayer full-thickness skin substitutes.

Commercially available skin substitutes	Composition	Cellular composition	Use	Characteristics	References
Epidermal Substitutes					
Epicel®	Epidermal sheets	Autologous keratinocytes cultured in air-liquid interface	Superficial wounds, Burns	- Fragile and challenging for surgical manipulation surgical adult -Allogeneic keratinocytes are often rejected	Supp and Boyce (2005), Wood et al. (2006), Nathoo et al. (2014)
Dermal Substitutes					
Biobrane®/TransCyte®	Epidermal layer composed of a silicone membrane and bonded to a collagen-1 - coated porous nylon mesh, acting as a dermal layer	It can be pre-seeded with allogeneic neonatal fibroblasts	Early application onto clean partial thickness wounds such as autologous donor sites and burns	-Improved cellular adhesion - Improved healing properties thanks to the neonatal fibroblasts - Requires secondary intervention to remove silicone layer after granulaiton tissue deposition	Tavis et al. (1980), Hansbrough (1995), Noordenbos et al. (1999), Amani et al. (2006), Whitaker et al. (2008)
Dermagraft®	PGA	Neonatal allogeneic fibroblasts	Chronic wounds and Diabetic Foot Ulcers (even in weight-bearing patients)	-Cryopreserved-Permanent dermal replacement -Donor cells are eventually lost -New epidermis grows from the wound edge -Used with a meshed STSG on full thickness wounds	Gentzkow et al. (1996), Marston (2004)
AMERIGEL®	Polyethylene glycol (400 and 3,350) and Oakin (antimicrobial)		Venous insufficiency ulcers, Pressure injuries stage 1–4, Diabetic skin ulcer	-Maintains moisture -Liquifies at body temperature, improving migration to wound site - Antibacterial	Jonathan Moore (2008)
AlloDerm®	Cryopreserved cadaveric human decellularised dermis		Burns, dermal extracellular scaffold Breast reconstruction Soft tissue augmentation around teeth implants, gingival augmentation, root coverage, graft protection and containment	-Allows fibroblast infiltration -Highly angiogenic -Can be meshed to improve epidermal perfusion -Used with a STSG can act as i full thickness	Wainwright (1995), Buinewicz and Rosen (2004)
OASIS®	Matrix derived from swine jejunum submucosa		Burns, Pressure ulcers, venous ulcers, Trauma wounds (abrasions, lacerations, second-degree burns, skin tears), post-Mohs surgery, podiatric, wound dehiscence	-Acts as scaffold for wound closures -Facilitates fixation and preservation os staples and sutures	Límová (2010)
Orcel®	Type I bovine collagen sponge	-fibroblasts -keratinocytes	Burns, Skin Ulcers	-stimulates host cell migration Matrix degradation takes 2–3 weeks	Shores et al. (2007), Shevchenko et al. (2010)
Elasto-Gel™	Glycerin-based gel merged with a hydrophilic polymer	N/A	Pressure ulcers, Diabetic wounds, first- and second-degree burns	-High absoprtion capabilities of wound exudate -Glycerin acts as antimicrobial	(Stout and McKessor, 2012)
EZ Derm®	Porcine derived xenograft with collagen-aldehyde crosslink	N/A	Burns, Ulcers, Partial thickness	-Prevents protein loss -Supports granulation tissue development	Troy et al. (2013)
Matriderm®	Multiparous three-dimensional matrix made from bovine collagen types I, III and V, and elastin	N/A	Modulates scar tissue formation, Burns, Chronic Wounds	-Haemostatic properties -Minimise the risk of split skin sub-graft haematoma -High biocompatibility	Cervelli et al. (2010)
Bilayer full thickness Substitutes					
Apligraf®	Bovine collagen 1	-Allogeneic neonatal fibroblasts -Neonatal keratinocytes	Chronic ulcers and deep wounds	-Supports angiogenesis (formation of new blood vessels), collagen production, and epithelialization -FDA approved	Edmonds, (2009)
Integra®	Cross-linked collagen scaffold with glycosaminoglycans (GAGs) overlaid with silicone as epidermal replacement	N/A	Burns where autograft may not be available at the time of excision, repair of scar contractures, treatment of partial and full-thickness neuropathic diabetic foot ulcers, trauma wounds	-After 6 weeks, silicon layer is replaced by a STSG or epidermal autographs -Tehcnically challenging and exoensive	Heimbach et al. (2003), Weigert et al. (2011)
Endoform®	Xenograft extracellular matrix scaffold	N/A	Surgical trauma, partial and full-thickness wounds, Ulcers	-Supports cell infiltration -Supports tissue regeneration	Magin et al. (2016)
Epifix®	Multilayered allograft composed of: epithelial lining amnion chorion, collagen connective tissue, growth factors cytokines	N/A	Burns, Diabetic Foot Ulcers	-Reduced protein/electrolyte loss -Supports healing	Zelen et al. (2013), Carruthers et al. (2015), Ilic et al. (2016)
Allomax®	non-crosslinked human dermis sheet		Chronic Burn wounds, Breast Reconstruction	-Acts as scaffold for new tissue deposition	Carruthers et al. (2015)
DermaPure®	Decellularised dermal allograft (human donor skin)	N/A	Chronic wounds, Ulcers, Traumatic Injuries	-Intact vascular-like channels to support angiogenesis and endothelial cell proliferation	Kimmel and Gittleman (2017)
Graftjacket®	Decellularised dermal allograft (human donor skin)	N/A	Ulcers, Full thickness wounds	-Supports cell migration into the grafts -Highly angiogenic	Brigido, (2006)

The epidermal layer plays a role in wound closure and acts as a barrier. It can be transplanted onto a wound bed that is capable of accepting it, such as in a split-thickness skin graft (STSG). Epidermal replacements produced in a laboratory setting can be either a single layer of keratinocytes cultivated in a combination of air and liquid and then transformed into a layered structure for transplantation, such as the commercially available Epicel (Table 1) or a solution of keratinocytes that is sprayed over the wound. Although keratinocytes can replace the several layers of the epidermis, other critical cell types are lacking such as melanocytes and immune cells. The use of epidermal substitutes in sheet form and as sprayed on cells is still limited for these reasons. Biodegradable matrices such as fibrin and hyaluronic acid are under development as delivery to support the fragile epidermal sheet (Baier Leach et al., 2003). In deeper wounds, the epidermal layer can be placed on top of a dermal substitute.

Dermal replacements serve as alternatives to granulation tissue, providing a surface for the growth of epidermal cells in order to facilitate wound closure. Dermal substitutes primarily comprise of decellularized ECM (AlloDerm^®^ or OASIS^®^, Table 1) or the structural proteins that make up the ECM (like collagen-based substitutes such as Orcel^®^, Matriderm^®^ and Apligraf^®^, Table 1). Alternatively, some dermal substitutes are partly or completely formulated with synthetic compounds like silicone (Biobrane^®^, Table 1) or PGA (Dermagraft^®^ and AMERIGEL^®^, Table 1). These dermal substitutes can be transplanted either as acellular scaffolds or with the addition of fibroblasts, the primary cells responsible for depositing and restructuring the ECM (Kim and Jacobs, 1996; Azagarsamy and Anseth, 2013).

Full-thickness skin substitutes consist of two layers, with one layer serving as an epidermal substitute and the other layer serving as a dermal replacement. The interaction between epithelial and mesenchymal cells has been demonstrated to enhance the structural integrity of artificially generated skin replacements in a laboratory setting (Lupu et al., 2023). A study conducted by Wojtowicz et al. revealed the anatomical and mechanical advantages of a coculture platform where fibroblasts and keratinocytes communicate (Wojtowicz et al., 2014). This has been further discussed by other authors (Dimatteo et al., 2018).

Bilayered substitutes are designed with the goal of replicating natural skin and enhancing the healing process. The bilayered skin substitutes are highly sophisticated treatments for wound healing and offer significant benefits for the majority of chronic wounds (Mariani et al., 2019) Nevertheless, with a thickness of around 2.5 mm, these alternatives rely on early vascularisation in order to achieve a successful graft take, as the maximum distance for oxygenation diffusion is around 0.2 mm (La Manna et al., 2021). In addition, full-thickness skin replacements are often accompanied by a substantial price tag. Therefore, researchers have investigated several combinations and preparations, such as including specific cells beforehand, in order to attain cost-effective and desirable medical results (Alina et al., 2020; Zhang X. et al., 2020; Zhang et al., 2018a).

Commercially available bilayered full thickness skin substitutes are often derived from decellularised scaffolds rather than bioengineered tissue layers (such as Epifix^®^ or DermaPure^®^, Table 1), limiting concerns of biocompatibility and simplifying the manufacturing process.

### 3.1 Origin of cellular components of commercially available skin substitutes

Cells utilised in both dermal and epidermal substitutes might originate from either autologous or allogeneic origins. Autologous cells are more advantageous in this aspect, but the need to cultivate them outside of the body before transplantation makes the process time-consuming and costly, and the negative effects on the donor site are an additional constraint.

Alternatively, allogeneic cells have the capability to be pre-seeded and cryopreserved in a skin substitute that is readily available for usage (Pineda-Castañeda et al., 2023). Nevertheless, allogeneic cells necessitate thorough examination to minimise the likelihood of infection and are ultimately expelled from the graft as a result of immunological rejection. Nevertheless, they remain in the skin for a sufficient duration to allow host cells to replenish the layers of the skin as they undergo turnover and modify the implanted material.

Neonatal allogeneic cells are more effective because they have a greater ability to multiply, and their HLA markers are not well-formed (Halim et al., 2010; Kennedy et al., 2011). The dermal fibroblasts utilised in skin substitutes are consistently of the papillary type, originating from the superficial layer. These fibroblasts exhibit a higher ratio of collagen III to collagen I compared to the deeper reticular fibroblasts (Van Vlierberghe et al., 2011). This ratio is closer to the one observed in the healing wound. Research has demonstrated that adult allogeneic fibroblasts can be accepted by the host tissue for a period of 2 months before being progressively substituted by host cells (Van Vlierberghe et al., 2011). The advantages and drawbacks of existing skin substitutes are examined here, with a focus on biological dermal substitutes.

### 3.2 Limitations of skin substitutes

The current skin substitutes in use have various drawbacks, such as the requirement for multiple operations, the fragility of grafts, inadequate engraftment, the necessity for autologous/allogeneic cells, and the need for surgical implantation into deeper and irregular wounds. The requirement for several surgeries primarily arises from the necessity to create viable granulation tissue before wound closure (Zhang X. et al., 2020).

Several well-researched biomaterials, including alginate, hyaluronic acid, PLGA, agarose, and chitosan, are recognised for their non-/low-antigenic properties. These materials have the ability to engage with the immune system and provoke a multifaceted response. Efforts have been undertaken to decrease the immune response to these biomaterials by adjusting their biochemical characteristics and modifying their surface features. Nevertheless, they may still elicit the typical foreign body reaction in the host (Li and Mooney, 2016; Lupu et al., 2023).

Our research team has demonstrated that variations in manufacturing procedures, including decellularisation and crosslinking protocols, can modify the physical properties of natural decellularised bilayered skin replacements. These modifications can potentially influence the clinical consequences of utilising these substitutes. Several investigations have indicated that the cross-linked ECM-derived scaffolds exhibit reduced cellular infiltration and impaired vascularisation (Dimatteo et al., 2018; Kim et al., 2019; La Manna et al., 2021). In order to overcome these constraints, researchers have proposed the use of hydrogels in tissue engineering. Hydrogels are advantageous because they are highly permeable to cells and can effectively transport therapeutic chemicals, growth factors, and cells necessary for the healing process.

## 4 Hydrogels developed by modified bioengineering techniques

Hydrogels are biomaterials made of polymers with a porous three-dimensional structure and can hold up to 99% of their weight in water. These hydrogels may be easily shaped into irregular tissue compartments to promote tissue regeneration and have demonstrated encouraging results in wound care (Ifkovits and Burdick, 2007; Alina et al., 2020).

An optimal hydrogel should replicate the mechanical characteristics of the surrounding tissue, enabling even distribution of stress and adhesive properties that facilitate the integration of the graft. These qualities are a result of the chemical composition of the polymers that make up the material. An optimal polymer for hydrogel design should possess the following indispensable characteristics: (i) The hydrophilic character of the material enables it to interact with physiological fluids for metabolic exchange. (ii) The material’s ability to adhere to surrounding tissue through mechanical or chemical interlocking facilitates the infiltration and migration of cells through the ECM. (iii) Biocompatibility to avoid the production of toxic substances or immune responses, (iv) Flexibility to shape into irregular wound forms, (v) Biodegradability to enable constructive remodelling and differentiation of the necessary cell types. After the hydrogel is implanted into a cleaned wound, the biomaterial breaks down and is replaced by a new matrix that is suitable for the specific area of the body. This new matrix is formed by the healthy cells that have moved into the wound from the surrounding tissue or the edge of the wound. Collagen and glycosaminoglycan-based biomaterials have been demonstrated to facilitate the restoration of vertebral disc height by infiltrating cells and promoting the regeneration of the appropriate ECM (Kim and Jacobs, 1996; Azagarsamy and Anseth, 2013; Pineda-Castañeda et al., 2023). These biomaterials are also utilised in other tissue-specific regenerative strategies. The remodelling phase in all tissues is influenced by both mechanical cues and the biochemical features of the designed graft, particularly when it is seeded with stem cells (Halim et al., 2010; Kennedy et al., 2011; Van Vlierberghe et al., 2011).

In addition to imitating mechanical properties, the hydrogels must possess adequate porosity to accommodate cells and interconnectivity to facilitate the efficient flow of nutrients and oxygen. Additional desirable characteristics of hydrogels for wound healing include antibacterial qualities and elasticity to provide support for wounds in places with joints (Miranda-Calderon et al., 2022).

Hydrogels can consist of either naturally derived or manufactured polymers or a combination of both. Natural polymers typically consist of sugars or amino acids, but synthetic polymers provide greater chemical diversity, repeatability, and tunability than natural polymers (Park, 1999). Natural polymers are present in biological tissues and organs, and they mimic the structure and composition of the ECM (Table 2). On the other hand, synthetic polymers are composed of organic molecules that can interact with cell surface receptors and biological macromolecules such as proteins (Shi et al., 2006; Garbern et al., 2011; Cho et al., 2017; Chen et al., 2023). This paper will explicitly examine the procedures involved in fabricating hydrogels, which are similar to those used in other biomaterial designs. In general, the design of hydrogels involves two main steps: selecting the polymers that make up the hydrogels, and the gelation process that gives the hydrogels their unique features.

**TABLE 2:** Advantages and disadvantages of atural polymers used in hydrogels.

Polymer	Advantages	Disadvantages
Collagen	Biocompatible, promotes cell growth	Expensive, potential for immune response
Chitosan	Antimicrobial, biocompatible	Limited mechanical strength solubility issue
Alginate	Biocompatible, easy to gel	Poor cell adhesion, requires cross-linking
Gelaline	Biocompatible, supports cell adhesion	Rapid degradation, thermal instability
Hyaluronic Acid	Biocompatable, promotes wound healing	High cost potential for rapid degradation

### 4.1 Polymers used in the production of hydrogels for wound healing

**FIGURE 2:**
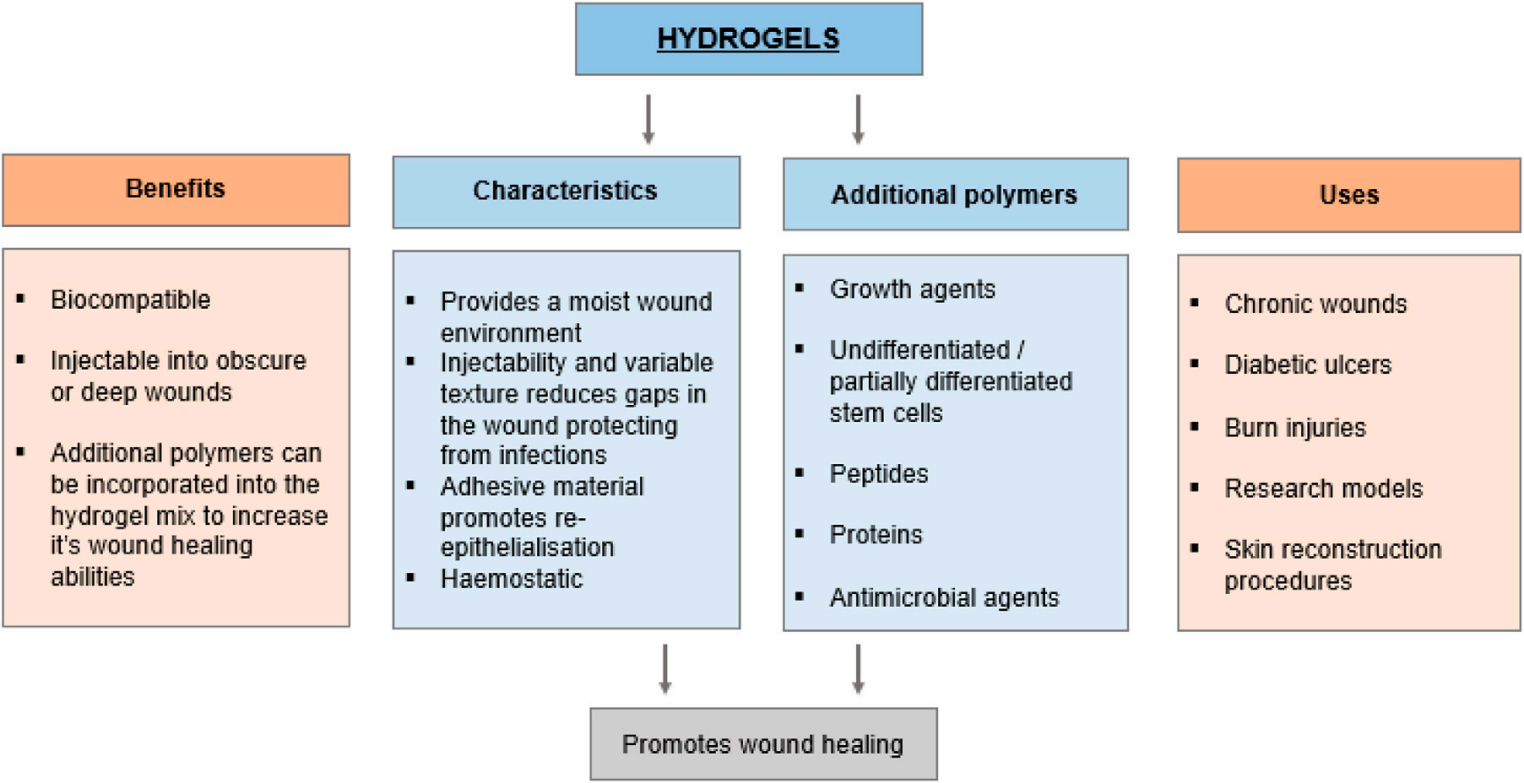
Characterisation of hydrogels for healthcare application.

Polymeric hydrogels have been extensively studied in tissue engineering and biomaterials, utilizing both natural and synthetic polymers. Their popularity stems from their versatile properties, which allow them to closely replicate the extracellular matrix (ECM) in terms of biocompatibility, biodegradability, water retention, and ability to support cells during tissue regeneration (Zhu and Marchant, 2011). These materials are typically made from hydrophilic polymers that form networks through either physical interactions (non-covalent bonds) or chemical cross-linking (covalent bonds), creating insoluble structures (see Figure 2). Synthetic polymers offer notable advantages, such as chemical variety and mechanical adjustability. They can be precisely engineered to imitate the complex, fibrous architecture of the ECM in biological tissues. Examples of such polymers include polyethylene glycol (PEG) and polyesters like polyglycolic acid (PGA), polylactic acid (PLA), and polycaprolactone (PCL) (Cho et al., 2017).

Hydrogel polymers that are artificially created can be designed to mimic the mechanical and biological characteristics of the missing ECM. These polymers can be easily replicated in large quantities without compromising their quality (Cho et al., 2017). Nevertheless, they do not possess the biological signals found in the natural polymers extracted from the ECM and may contain leftover toxins from the post-processing stage (Chatterjee et al., 2018). In addition, numerous synthetic polymers exhibit rigidity and are non-biodegradable, which restricts their potential use in soft tissue rebuilding or in conforming to unusual wound shapes. However, non-degradable polymers could potentially be used for replacing the epidermis in cases where there is a lack of an adequate barrier function, such as in burn wounds. Currently, there is a growing trend in creating synthetic polymers to break down within a specific timeframe. This significantly expands the potential use of these types of polymers in tissue engineering, especially in the development of hydrogels (Wang et al., 2017).

Due to their non-native nature, synthetic polymers have not been widely used in the body. As a result, hydrogels made from natural polymers have gained significant interest in recent years. Several natural polymers are obtained from sustainable biomass, specifically polysaccharides like cellulose, chitin, chitosan, alginate, carrageenan, xanthan gum, or dextran. Additional examples of polysaccharide-based polymers are hyaluronic acid and chondroitin sulphate. Protein-based natural polymers include collagen, gelatin, and fibrin. As previously stated, researchers frequently incorporate additional therapeutic compounds or cells into the hydrogel to ensure the continuous release of these therapeutic agents and attract necessary cell types to the wound area.

Depending on the polymer composition of the hydrogels, they can be classified in pure, hybrid or composite hydrogels. Pure hydrogels are made from a single natural or synthetic polymer network, which results is well-characterised biomechanical properties that can be exploited for different uses. For instance, pure hydrogels are already been used in other clinical areas, such as breast reconstructions or for drug delivery applications (Radwan et al., 2017; Franceschini, 2021). As pure hydrogels are often made with known biomaterials, they also favour regulatory approval and commercialisation, making them readily available for the clinic. In addition, the relative simplicity in single-polymer hydrogel construction is an advantage for large-scale manufacturing. In the context of wound healing, single native polymers like collagen (Jridi et al., 2015; Ge et al., 2020; Lu et al., 2020) or alginate-based (Lu et al., 2020) hydrogels present high biocompatibility and promote ECM remodelling both *in vitro* and *in vivo.* In fact, collagen-based scaffolds are commercially available in the form of Nevertheless, single component hydrogels typically present poor mechanical properties which cannot be tailored for specific physical and chemical requirements, resulting in low versatility and limited extrapolation for different types of clinical wounds (Asadi et al., 2021).

Nowadays, most studies focus on the development of composite or hybrid hydrogels able to combine the properties of various biomaterials to maximise the wound healing effect. Composite hydrogels are characterized by the addition of other biomolecules to the polymer gel network to enhance the mechanical and bioactive properties of the hydrogel. (Shang et al., 2024). In 2021, a team in China created silica-based nanocomposite hydrogels that are bioactive and antibacterial. These hydrogels showed improved angiogenesis. The primary structure of the nanocomposite hydrogels consisted of polyethene glycol diacrylate combined with bioactive glass nanoparticles containing copper and sodium alginate. Within a controlled laboratory environment, the hydrogel greatly stimulated the growth and ability to form blood vessels of endothelial progenitor cells. In a deep diabetic wound, it repaired the network of blood vessels by increasing the expression of VEGF and the deposition of collagen matrix (Li et al., 2020). As a result, it significantly sped up the formation of granulation tissue and the healing of the wound.

Alternatively, hybrid hydrogels constructed as a combination of different polymer networks at the hydrogel core enable the mechanical properties of the gel to closely resemble the ones in native skin (Cao et al., 2023; Yang et al., 2023).

Recently, the advantages of both hybrid and composite hydrogel have been exploited to construct all-in-one skin-like hydrogels with low cytotoxicity, able to promote cell proliferation and ECM deposition and with angiogenic and antimicrobial properties (Yang et al., 2022; Bakadia et al., 2023; Chen et al., 2023; Mahheidari et al., 2024). For instance, in 2022, Yang and colleagues developed a multi-functional hydrogel that induced wound healing 25% faster that other commercially available wound dressings, exhibiting limited immune activation and no infections across the 14 days of treatment. They combined the mechanical properties of synthetic PEG with the natural-derived polymer chitosan and they integrated functional antibacterial compounds like methacrylamide dopamine and ion zinc (Yang et al., 2022). In a similar approach, Bakadia’s group improved the poor mechanical properties of silk sericin (SS), an organic polymer known for its cell proliferation and collagen deposition stimulation capacities, crosslinking it with synthetic PVA (poly vinyl alcohol). As in Yang’s paper, they also enhanced the antimicrobial properties of the gel by adding the antibiotic and antifungal azithromycin (AZM). Once again, the hybrid composite SS/PVA/AMZ hydrogel promoted wound healing *in vivo* to an 100% wound close rate at day 24, compared to a 60%–75% in the same timepoints for the control and commercially available dressing groups (Bakadia et al., 2023).

### 4.2 Hydrogel production process

**FIGURE 3:**
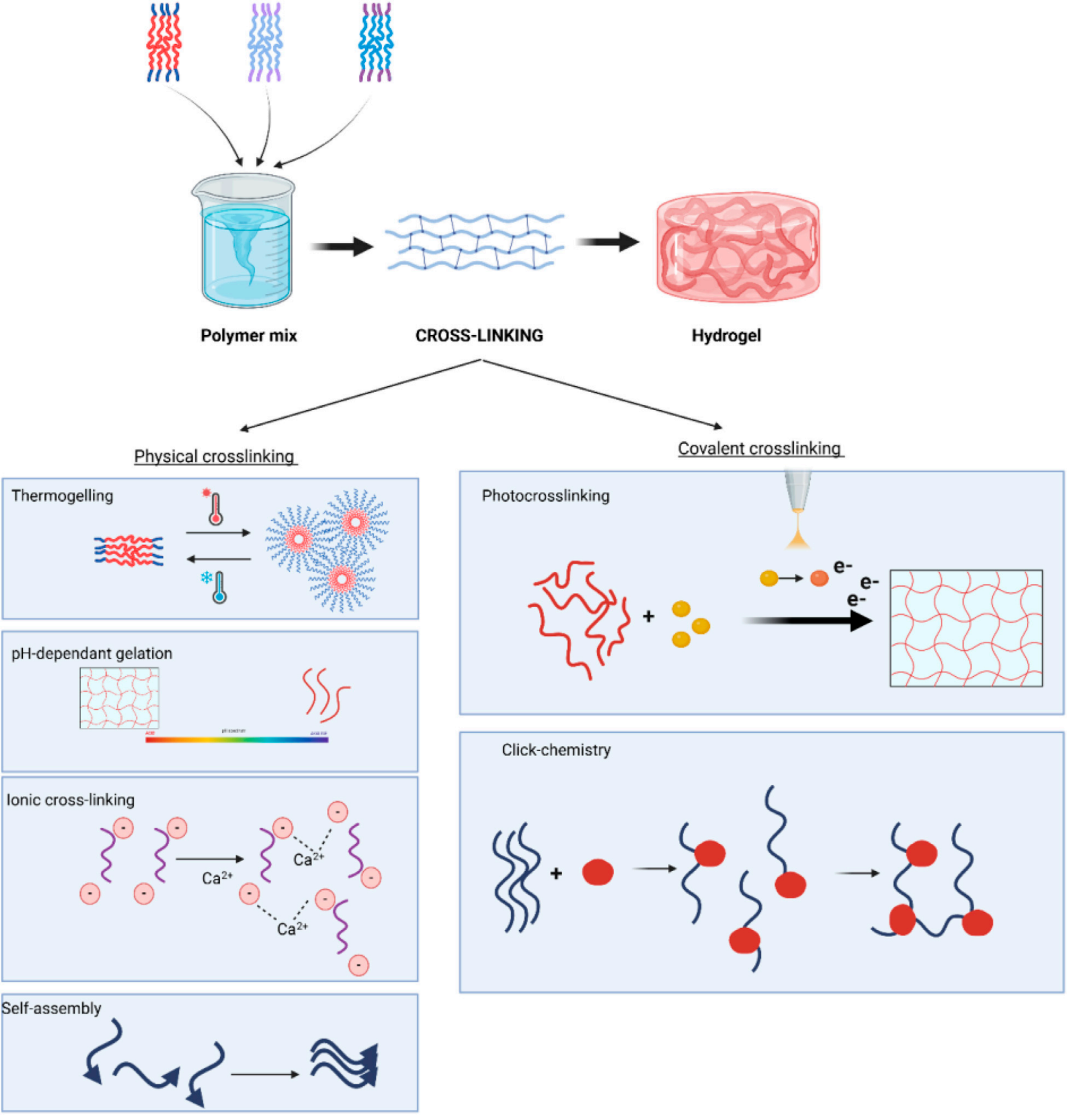
Schematic representation of hydrogel formation, highlighting covalent crosslinking for direct gelation or mechanical reinforcement of physically cross-linked hydrogels with tuneable properties.

The assembly of polymers, whether they are natural or synthetic, to create hydrogels is a crucial determinant of hydrogel functionality. Furthermore, this process might have a subsequent impact on the outcomes of healing. In recent times, several techniques have been developed to create micro- or nanogels with precise control over their topography, degradation, shape, size, and mechanical properties, resulting in optimal outcomes Table 3. The techniques can generally be classified reversible physical cross-linking techniques and covalent cross-linking reactions, as summarised in Figure 3.

**TABLE 3:** Characteristics, advantages and disadvantages of the physical and chemical polymer crosslinking methods for hydrogel production.

Method	Physiochemical	Mechanical	Biological	Advantages	Disadvantages
Physical crosslinking	-Non-toxic -Reversible bonding	Lower mechanical strength	Good bio-compatibility	-No toxic crosslinkers are required -Easy to process	-Weak mechanical properties -Sensitive to environmental conditions (e.g., pH, temperature)
Thermogelling	Temperature-dependant properties	Variable strength	Non-toxic (depending on materials)	-Self-gelling at physiological conditions -Injectable	-Limited by temperature range -May need additional crosslinkers
Ionic crosslinking	Variable gelation via ionic interactions	Moderate mechanical strength	Good for drug delivery	-Mild conditions -Responsive to environmental stimuli	-Lower stability -Limited mechanical strength
Self-Assembly	Self-assembling peptides	Lower mechanical strength	High biocompatibility	-Minimal chemical inputs -Mimics natural assembly processes	-Poor mechanical properties -Limited to specific biological conditions
Chemical crosslinking	Stable structure	High mechanical strength	Possible toxicity from reagents	-Strong, durable hydrogels -Control over network density	-May require toxic crosslinkers -Residual chemicals may limit biological applications
Photopolymerisation	-Rapid -Spatio temporal control	Good mechanical properties	Can be cytotoxic depending on photo-initiators	-High control over gelation -Can pattern structures	-Photoinitiator toxicity -Requires UV light, which may damage cells
Click-chemistry	-High specificity -Rapid reaction	Good mechanical properties	High biocompatibility	-Fast, efficient reaction -High biocompatibility	-Requires specific reagents -Can be costly

#### 4.2.1 Hydrogel production process through reversible physical interactions

Polymer solutions undergo a sol-gel transition, which is influenced by physical factors like temperature, pH, and intermolecular binding forces. These physical forces possess inherent reversibility and hence must maintain stability at the location of hydrogel implantation. In addition, physical hydrogels exhibit worse mechanical strength compared to covalently cross-linked hydrogels (Zhang X. et al., 2020). The mechanical and physical factors that drive physical cross-linking include temperature, pH and ionic and non-covalent forces.

##### 4.2.1.1 Thermogelling hydrogels

Thermogelling hydrogels are composed of amphiphilic polymers that contain both hydrophilic (water-attracting) and hydrophobic (water-repelling) domains. These polymers exhibit solubility at ambient temperatures, but upon exposure to elevated temperatures, such as body temperature (approximately 37°C), they undergo a phase transition (Liow et al., 2016). The hydrophobic segments self-assemble into micellar structures, while the hydrophilic domains are exposed to the surrounding aqueous environment. This temperature-responsive behaviour allows for the controlled gelation of the hydrogel in response to body temperature, making them ideal for use in biomedical applications. A notable example of thermogelling hydrogels are Pluronics, which consist of triblock copolymers that display the characteristic temperature-sensitive sol-gel transition. These hydrogels are particularly useful in drug delivery systems, as they can encapsulate both hydrophilic and hydrophobic therapeutic agents. Upon gelation, they provide sustained release of the drug over time, offering an effective approach for creating long-term drug depots (Li and Mooney, 2016; Jang et al., 2019; Lupu et al., 2023).

However, a limitation of thermogelling hydrogels in cutaneous (skin) applications is their sensitivity to lower temperatures. When exposed to colder environments, these hydrogels may experience destabilization or premature gel breakdown. Therefore, it is crucial to design thermogelling systems with a Lower Critical Solution Temperature (LCST) that is close to ambient temperatures, ensuring stability during application and minimizing the risk of gel disruption upon skin contact (Gong et al., 2013; Khan et al., 2023).

##### 4.2.1.2 pH-dependent gelation

pH-responsive gelation leverages the ability of functional groups within polymeric constituents to donate or accept protons, facilitating controlled hydrogel formation. This mechanism is particularly useful in environments with stable pH to maintain hydrogel integrity, but it can also be strategically utilized for pH-triggered therapeutic drug release, especially in pathological conditions where local pH fluctuations occur (Kim et al., 2019). Several pH-sensitive polymers have been explored for biomedical applications. Chitosan, for instance, undergoes gelation in mildly acidic conditions due to protonation of amine groups, making it highly suitable for wound healing applications. Poly (acrylic acid) (PAA) and poly(N-isopropylacrylamide) (PNIPAM) are also well-known for their pH-dependent sol-gel transitions, often used in drug delivery and tissue engineering. Additionally, alginate-based hydrogels crosslink at neutral and slightly acidic pH when exposed to divalent cations like Ca^2+^, further broadening their application in bioengineering (Zhang Z. et al., 2018; Vegad et al., 2023).

##### 4.2.1.3 Ionic cross-linking hydrogels

Ionic cross-linking hydrogels are formed through the interaction between polymers and multivalent counterions, such as calcium ions, which create ionic bonds and salt bridges between polymer chains (Parhi, 2017). These cross-links lead to the formation of a three-dimensional gel network. The mechanical properties of ionic hydrogels can be controlled by varying the concentration of both the polymer and the counterion, offering a tunable approach for optimizing hydrogel stiffness and flexibility.

An exemplary system for ionic cross-linking is the formation of alginate hydrogels, where alginate polymer chains are cross-linked with calcium chloride to form a stable gel (Wang et al., 2025). The concentration of calcium chloride, along with the alginate concentration, plays a significant role in determining the final mechanical properties of the hydrogel. This tunability makes ionic cross-linked hydrogels suitable for a variety of biomedical applications, including wound dressings, tissue engineering, and drug delivery (Zilberman et al., 2015).

One key advantage of ionic cross-linking is the ability to achieve rapid gelation, which is critical for *in situ* applications where the gel must form quickly at the target site. For instance, alginate gels typically undergo gelation within 30 min when calcium chloride is added, making them ideal for scenarios where fast and localized gel formation is required. This rapid gelation time is particularly advantageous in applications such as injectable drug delivery or the formation of tissue scaffolds (Dimatteo et al., 2018; Wang et al., 2025).

##### 4.2.1.4 Intra-molecular self-assembly

Many proteins and peptides possess domains specifically evolved to bind complementary peptide sequences, leading to self-assembled hydrogel networks. When multiple binding sites are present, such as in polar peptide sequences, extensive supramolecular structures can form upon mixing, driven by various non-covalent interactions (Braun et al., 2020; La Manna et al., 2021; Sedighi et al., 2023) The forces promoting gelation include hydrogen bonding, electrostatic interactions (ionic bonding), and hydrophobic interactions, which collectively contribute to network stability. Notably, β-sheet-forming peptides, such as RADA16-I, self-assemble into nanofibrous hydrogels under physiological conditions, demonstrating significant potential for regenerative medicine. Furthermore, peptides can be chemically conjugated to high-affinity binding pairs like biotin-avidin systems, further enhancing crosslinking efficiency and hydrogel stability (Arosio et al., 2012; Alina et al., 2020; Genové et al., 2022).

#### 4.2.2 Hydrogel production process through covalent cross-linking

Hydrogel polymers can also be covalently cross-linked to form mechanically stable and irreversible bonds, as the primary cross-linking of the polymers or to reinforce physical crosslinks. For *in situ* covalent cross-linking, it is important that the reactions are carried out under mild physiological conditions. Over the years, a number of reaction types have been defined to meet this requirement. These include photoinitiated cross-linking and click chemistry.

##### 4.2.2.1 Photo cross-linking

Small molecule photoinitiators can be activated by specific wavelengths to induce light-mediated polymer cross-linking through a process known as photoinitiation. Upon activation, these initiators generate free radicals that propagate a chain reaction through unsaturated bonds in the polymer backbone, forming covalent crosslinks and stabilizing the hydrogel. The intensity of light exposure and polymer concentration can be adjusted to fine-tune the mechanical properties of the final hydrogel. Commonly used small molecule photoinitiators include Irgacure 2,959 (Nguyen et al., 2019; Tomal and Ortyl, 2020), widely employed for UV-mediated hydrogel crosslinking due to its biocompatibility, and lithium phenyl-2,4,6-trimethylbenzoylphosphinate (LAP) (Tomal and Ortyl, 2020; Tomal et al., 2024), which is effective under visible light, minimizing potential cytotoxic effects. Camphorquinone is another widely used initiator, particularly in dental and biomedical applications. Using wavelengths beyond white light, such as UV (typically 365 nm) or visible light (400–500 nm), allows precise control over polymerization kinetics. While UV-based photopolymerization remains the most common approach, alternative wavelengths are being explored to optimize hydrogel properties and reduce surface thrombogenicity in crosslinked materials (Kim and Jacobs, 1996; Ifkovits and Burdick, 2007; Scheideler et al., 2007; Tomal and Ortyl, 2020).

##### 4.2.2.2 Click-chemistry

This refers to a type of reaction between chemical groups that is instantaneous and selective under mild conditions, making it an attractive approach for *in situ* hydrogel formation under physiological conditions. The reactions take place in aqueous solutions and generate minimal toxic waste products. Click chemistry, which includes a number of reaction types such as alkyne-azide click, Diels–Alder click, thiol-ene click, thiol-ene click and thiol-Michael addition reactions (Kolb et al., 2001; Azagarsamy and Anseth, 2013). They allow scalable and efficient cross-linking of the reactants. Of these reaction types, the Cu^2+^ catalysed alkyne-azide click chemistry has been the most widely used (Pineda-Castañeda et al., 2023) and has led to issues with residual Cu^2+^ toxicity when incorporating live cells or applied *in vivo* (Kennedy et al., 2011).

### 4.3 Biomimetic hydrogels incorporating extracellular matrix

Biomimetic hydrogels, sometimes referred to as ECM-based hydrogels, consist of bioactive materials, such as the ECM or its components, that are included in their composition. The ECM is the structural component found in all tissues and organs that offers the essential mechanical support needed for tissue development and specialisation (Kennedy et al., 2011). Usually, the ECM that is included in hydrogels goes through a series of stages to eliminate cellular material and waste through a decellularisation process (Figure 5). The residual scaffold material maintains the structural ECM and the chemical signals that facilitate cell adhesion and movement, hence and enabling the infiltration and differentiation of host cells (Halim et al., 2010).

**FIGURE 4:**
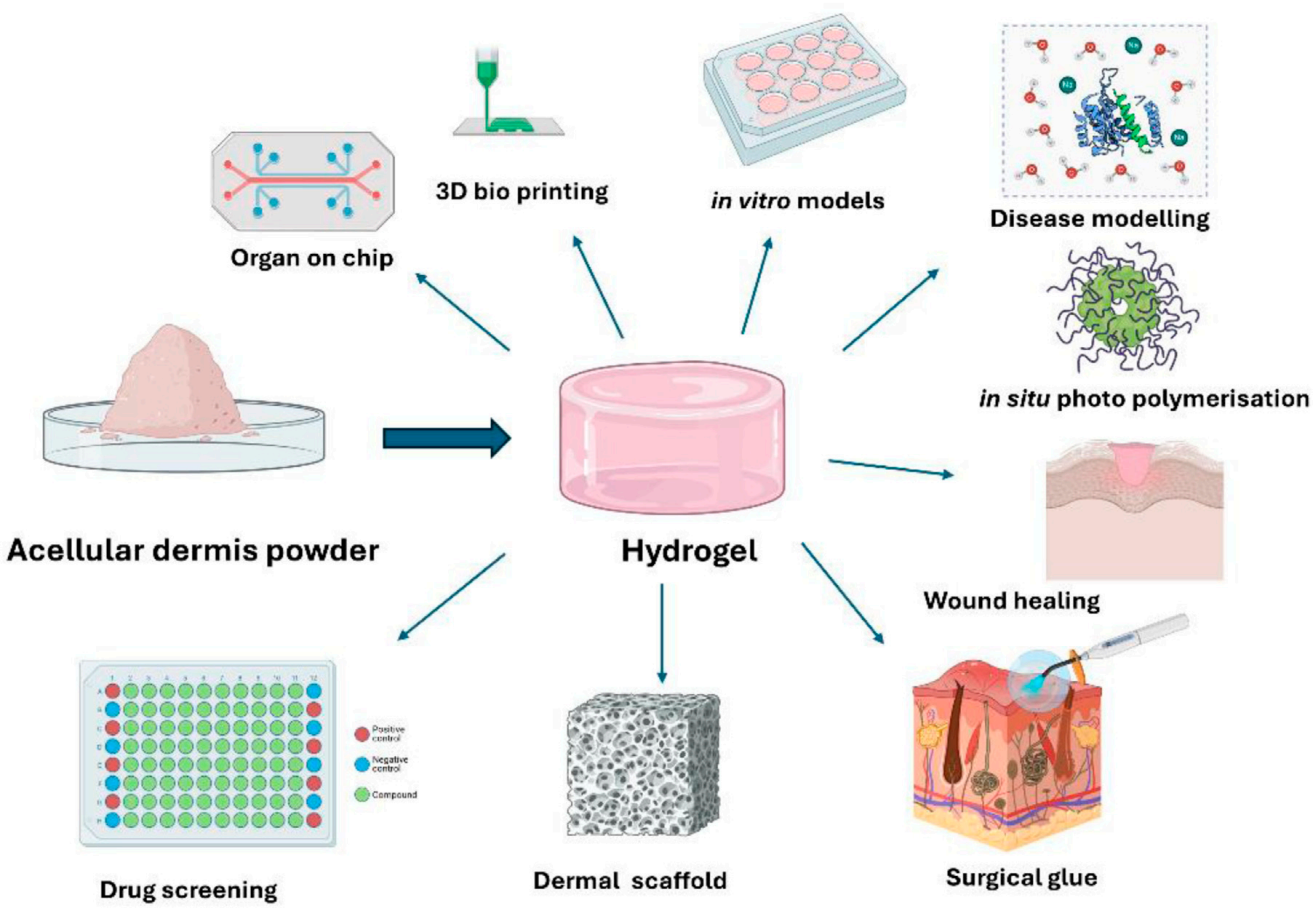
*In vitro* applications of biomimetic ECM-derived hydrogels.

The structural composition and resemblance to genuine soft tissues make this hydrogel highly promising for tissue engineering applications (Figure 4), yet it may suffer from a lack of mechanical stability. Therefore, it can be chemically bonded to enhance surgical manipulation and prolong their breakdown process in the body. The penetration of cells into highly crosslinked hydrogels may be slower, leading to the activation of the body’s immune response. Nevertheless, the results are contingent upon the degree of crosslinking and chemical composition. Our group has been studying the process of improving the mechanical properties of biomaterials through chemical modification. We have specifically used genipin, a natural agent, as a mild crosslinking agent. This has resulted in a significant increase in mechanical strength compared to other chemicals, thanks to the specific chemical reactions that occur (Shi et al., 2006; Garbern et al., 2011).

Synthetic hydrogels generally exhibit a more intricate biocompatibility profile compared to hydrogels constructed of natural proteins. Efforts have been made to mitigate the immunological reaction by modifying the chemical composition and surface characteristics of the biomaterial. The objective is to minimise the adverse reactions that can be triggered by the artificial polymers (Chen et al., 2017; Cho et al., 2017). Polymers that are obtained from natural sources, like ECM, do not seem to cause the usual immune response that occurs when foreign substances are introduced into the body. Instead, they elicit a favourable natural and restructuring response linked to an adaptive immune response (Zhu and Marchant, 2011). It should be noted that if the decellularisation procedure does not effectively remove all the antigens from the ECM, the remaining cell fragments in the hydrogel may induce an immune response, leading to delayed healing. This phenomenon is known as a foreign body reaction.

Another consequence caused by a foreign object or chemical by-products that leads to an intensified inflammatory reaction is a delayed healing process, which can subsequently result in fibrosis. Consistently, the transplantation of acellular ECM-derived scaffolds appears to be controlled by an M2-like response, which results in reduced scarring and an ability to remodel in a beneficial way. This response supports the infiltration of host cells and promotes a regenerative environment. Successful regeneration relies on the constructive remodelling of implanted matrices. In order to simplify this process, multiple research teams have created hydrogels based on ECM (Huang et al., 2016; Dani et al., 2021). The objective is to enhance their effectiveness on uneven wound surfaces and to aid in the transportation of cargo cells.

Biomimetic hydrogels are best obtained from the same anatomical place to preserve the natural bioactivity of the tissue. However, it is important to ensure that the procedure employed to remove cellular material does not damage the biological cues needed for the remodelling process. The inherent mechanical qualities of the hydrogel, which is the end product, are determined by the anatomical source of the tissue from which the biomaterial is formed. Nevertheless, the elastic modulus of hydrogels differs from that of the decellularized ECM itself, allowing it to be customised to achieve the desired cellular response (Qin et al., 2022). Cellular differentiation and the synthesis of new ECM proteins are crucial processes for cells like fibroblasts, as they rely on mechanical signals for their functions.

The intricate biochemistry of decellularized matrices remains inadequately comprehended. Therefore, a more thorough analysis of the active constituents of the ECM will enhance the consistency of scaffolds and facilitate the standardised production of natural polymers. Additionally, by adjusting the synthetic polymers, it may be possible to alleviate immune rejection and achieve improved healing outcomes. This has been supported by previous studies (Chen et al., 2017; Dani et al., 2021).

### 4.4 Hydrogel design rationale for wound healing purposes

For wound healing applications, the manufacturing process of hydrogels must consider several factors to ensure optimal functionality of the scaffold. For instance, the scaffold must be able to faithfully mimic the mechanical structure and properties of the native skin to maintain tissue homeostasis across the injury site. As hydrogels in wound healing are often taking as a temporary approach, scaffold degradation must also be a tightly regulated process, which serves to the necessities of the wound. This is particularly important when designing multifunctional hydrogels combining the physical and mechanical properties of the scaffold with different bioactive elements.

#### 4.4.1 Viscoelastic properties

Skin is known to have viscoelastic properties which allow the tissue to recover its original shape after a force is applied to it. Unlike other purely elastic materials, skin does not store the energy of the force applied to them but is able to gradually release it through a phenomenon known as stress relaxation (Martinez-Garcia et al., 2021). This observed adaptability of the skin is not only protective against injury or physical stress, but it has been shown to have an important role in cell behaviour, migration and proliferation capacities. The capacity of ECM to absorb part of the initial stress and gradually recover its native state sends mechanical cues to the cell to dynamically adapt to the changes in their microenvironment and hence supporting tissue homeostasis (Li et al., 2024). Interestingly, a study published in 2021 showed that ECM-derived viscoelastic properties are organ-specific and it further supports the claim of ECM and its mechanic properties having an active role is supporting tissue function (Martinez-Garcia et al., 2021). Therefore, skin grafts must ensure they mimic the viscoelastic behaviour of the native ECM.

Typically, viscoelasticity can be measured based on the displacement from the original linear region after a force is applied (Young’s elastic modulus, SI unit = Pa) or after the angled displacement of the object after said force [shear stress modulus, SI unit = Pa). There has been a lack of consensus when characterising skin’s viscoelasticity, with results ranging from 5 to 17 kPa elastic modulus (Jachowicz et al., 2007), to other authors reporting data in the megapascal scale in the epidermis 3–12 MPa, (Kendall et al., 2007)]. It is widely accepted that the mechanic properties of dermal and epidermal layers change depending on tissue depth, hence the variability observed in published data could be explained by the specific probe lengths and measurements depth used in each study. Chrichton’s group in 2013 discovered that the measured Young’s moduli inversely correlated with the diameter of probed used, reporting data between 1 and 30 MPa. (Crichton et al., 2013).

Due to their high-water content and hydrophilic behaviour, hydrogels have intrinsic viscoelastic properties. Hydrogels, and particularly natural-polymer-derived hydrogels, have a moduli withing the1∼10^5^ Pa range that can be exploited to closely replicate those exhibited by native skin tissue (Li et al., 2024; Wang G. K. et al., 2024). Biomimetic and natural polymer-based hydrogels can be easily tuned to the desired viscoelasticity degree through changes in polymer composition (Hafeez et al., 2023) concentration (Nam et al., 2019; Patiño Vargas et al., 2022) and crosslinking characteristics and density (Bartnikowski et al., 2015; Li et al., 2024).

In the context of wound-healing, gelatin-based hydrogels have shown to faithfully emulate native skin viscoelasticity and trigger cell proliferation and skin regeneration. Recently, Wang et al. developed a conjugated-through-photopolymerization gelatin-hyaluronic acid (HA) hydrogel exhibiting a Young’s moduli between 20 and 140kPa, comparable to such of the human skin (Wang et al., 2023). By adjusting the gelatin:HA ratio, they were able to tune the viscoelastic properties of the gel. *In vitro* and *in vivo* data of the gel used as scaffold for a bilayer skin construct implanted in a murine model showed improved cell proliferation, adhesion and overall healing properties, confirming the tissue-regeneration capacities of the graft.

In this line of research, Xu et al., in 2021 were able to tailor the viscoelastic properties of a Gellan gum-gelatin based hydrogel by adjusting polymer ratio, achieving a consistent elastic (or compression) modulus of 14 kPa. Again, *in vitro* data of the hydrogel shows that it promotes fibroblast differentiation and, as such, stimulates skin regeneration (Xu et al., 2021).

Other groups have taken alternative approaches to apply viscoelastic properties of gels in skin wound healing. Plasma-derived hydrogels conjugated with agarose have been shown to have tuneable viscoelastic properties in concentration-dependant cross-linking process (Patiño Vargas et al., 2022). Although the group achieved up to 15 kPA elastic modelis at 2% agarose, which could be compatible with epidermal modulis as reported previously (Jachowicz et al., 2007), there was an observed time-dependant degradation of the viscoelastic network and it did not support long-term cell growth.

Synthetic polymer hydrogels with skin-like viscoelasticity have also been under development. Based purely on repulsion and attraction electromagnetic interactions, Sano’s and colleagues were able to develop a dynamic network of inorganic nanosheets of titanate and water inside the hydrogel. The nanosheets were able to repulse or attract each other in response to external stimuli, modifying the internal structure of the hydrogel and hence changing the elastic properties of the material, ranging between 26 and 600 kPa elastic modulis (Sano et al., 2020). Nevertheless, the applications of synthetic polymer-based gels in skin wound healing are yet to be defined.

#### 4.4.2 Degradation rate

Another factor to take into account when designing new hydrogels is the biological degradation rate. Gradual degradation of gels used as a scaffold to support native skin wound healing is an important element to consider when designing new hydrogels. This is particularly relevant in hydrogels used as drug-delivery systems or those releasing time-dependant growth factor and other bioactive compounds to stimulate tissue regeneration (Arabpour et al., 2024). A study published in 2020 by Thai et al. also suggested that hydrogel degradability is not only essential for scaffold removal after healing is completed and release of biological factors, but the degradation on its own also promoted seeded cell migration, proliferation and angiogenic capabilities (Thai et al., 2023). An interesting strategy is to rely on the biodegradability of polymers using native enzymatic machinery. During the inflammation stage after injury, damaged and immune cells release proteases such as matrix metalloproteinases (MMPs) and collagenases, which disrupt the ECM structure to allow cell migration to injury site (Thai et al., 2023). The use of natural-derived polymers like gelatin, hyaluronic acid or collagen present in native tissue ensures the gel can be degraded by intrinsic proteases; however this can pose as a challenge on the durability of the grafts due to its rapid degradation.

Efforts have been made to overcome the rapid degradation exhibited by biomimetic hydrogels. Zhao’s group in 2016 synthesised the common gelatin crosslinked with methacrylamide groups (GelMa hydrogels) at different concentrations to evaluate the degradation rate and were able to tune total degradation in the range of 3 days to up to 8 weeks, desirable for long term wound treatments (Zhao et al., 2016). Other studies have used the potential of the method to create tailored drug-delivery systems for different pathologies (Vigata et al., 2020). More recently, Rusu and colleagues in Basel developed a method to crosslink gelatin gels with nanocapsules able to protect the antibiotic cargo from biodegradation, which could undergo controlled-release upon pH changes (Rusu et al., 2023). Hyaluronic acid (HA)-based gels can also be tailored to ensure controlled release of bioactive compounds. In Ho et al., (2022), Zhou et al. used HA-PEG hydrogels embedded with antibacterial imidazolium ionic liquid during crosslinking to create wound dressings fully biodegradable in 24 h. *In vivo*, the gel network was disrupted by endogenous hyaluroneases at a constant rate, which allowed the controlled released of the antibiotic and other active substances, which overall guaranteed the sterility of the wound at 92% and boosted cell migration and vascularisation of the site of injury (Zhou et al., 2022).

Alternatively, synthetic polymers can also be chemically designed to be cleaved by native proteases (Thai et al., 2023). The biggest advantage of this approach is that the hydrogel degradation rate can be fully modulable by changing the concentration of the MMP-cleaved peptide added to the formulation.

#### 4.4.3 Pro-regenerative and immuno-engineered hydrogels

As the understanding of the interaction between the immune system and wound healing improves, more streamlined and direct immune-engineered and pro-regenerative approaches will emerge. The development of hybrid biomaterials has opened up the possibility of adapting their composition and enhancing their biocompatibility to improve tissue repair and regeneration within the host (Qin et al., 2022). It has been shown by our group that hybrid hydrogels exhibit more effective structural integrity and enhanced biocompatibility when acellular tissue was conjugated with polymers, such as PVA and alginate (Francis et al., 2018).

Incorporating functional groups, such as RGD; Arg-Gly-Asp, or small molecules, with anti-inflammatory and pro-angiogenic properties, into synthetic or natural polymers is another widely known strategy to improve the overall functionality of the hydrogels.

Xue and colleagues demonstrated that RGD domains conjugated with scaffolds promoted cell adhesion and improved healing (Xue et al., 2021). Another group coupled collagen I-hydroxybenzoic acid (COL-P) and hyaluronic-acid-tyramine (HA-Tyr) using horseradish peroxidase (HRP) to form COL-HA hydrogel (Ying et al., 2019). This hydrogel held a porous structure that facilitated the exchange of nutrients, other media, and gases. It was then injected into a full-thickness wound in mice, which resulted in the COL-HA hydrogel inducing a more complete healing compared to the individual components after 14 days, and a much better outcome when compared to the control (which was a commercially available wound healing formula called *Yunnan Baiyao*). Histological analysis showed that the use of the hydrogel restored epithelial coverage and provided a rich microenvironment for cell proliferation, differentiation and promotion of collagen and neovascularisation.

Other studies have demonstrated that the biochemical properties of the macromolecules incorporated into the hydrogels can alter the immunomodulatory properties of the scaffolds. Shen and colleagues showed that a sulphated, chitosan-based hydrogel reduced pro-inflammatory M1 macrophages and significantly improved diabetic wound healing (Shen et al., 2020). Another group showed that a dextran hydrogel modified with functional groups was able to regenerate complete skin structures with appendages in both acute wounds and pre-existing scars, as evidenced by an upregulation of M2 phenotype macrophages (Sun, 2017).

Biomaterial with immunomodulatory properties that has been successfully used in TE is the bioactive glass. Hydrogels incorporating bioactive glass have been reported to improve both hard and soft tissue regeneration, suggesting it is wide range of therapeutic uses (Zhu et al., 2020). Studies have shown that bioactive glass influences cell migration into the wound area, affects macrophage polarisation (Dong et al., 2017), and promote a pro-remodelling outcome by shifting macrophages from M1 to M2 phenotypes (Zhu et al., 2020).

To maximise skin repair, some authors have manipulated the malleability and texture of the hydrogel to promote cell migration and efficient delivery to the host wound (Yin et al., 2021). Meanwhile, others are using electrospinning technology to alter the topological structures of the biomaterial, which has been shown to increase monocyte recruitment and induce vessel formation (Gao et al., 2021). Additionally, some groups are working on strategies to reduce degradation by optimising the dosage of the crosslinking agents or using milder alternatives to support cell attachment (Greco et al., 2018), as well as optimising pore size to promote full-thickness skin regeneration (Sun et al., 2010; Sun et al., 2018).

#### 4.4.4 Incorporation of nanoparticles, growth factors and cells to hydrogels in wound healing

The potential for developing hydrogels that can be manipulated to carry out a range of functions is immense. Current clinical applications are centred around the *in vivo* use of hydrogels as carriers for bioactive molecules, growth factors, and nanoparticles, as well as a range of other biological materials in chronic wound healing.

One combination that is commonly used to aid wound healing is the incorporation of vascular endothelial growth factor (VEGF) and platelet-derived growth factor (PDGF) into hydrogels, thus producing a matrix possessing the dual function of acting as pro-regenerative scaffolds and accelerators of angiogenesis (Li et al., 2015; Siebert et al., 2021). Additionally, the use of fibroblast growth factors (FGFs) can promote collagen deposition aiding in regulating cell proliferation and differentiation during skin remodelling processes (Xiao et al., 2021).

Other groups have incorporated nanoparticles loaded with plasmid-encoded epidermal growth factor (EGF), due to its anti-inflammatory properties, to aid in the healing of infected wounds (Shao et al., 2021). Silver nanoparticles (AgNPs) have proven antimicrobial and healing properties that are safe for topical use at low concentrations (Kim et al., 2007; Durán et al., 2016; Oryan et al 2018). Diniz *et al.* successfully stabilised AgNPs by forming a hydrogel composed of natural polymers, sodium alginate and gelatine, which when combined act as a nucleation site by creating voids within the polymer network (Diniz et al., 2022). Studies have shown that the optimum ratio of sodium alginate and gelatine was suggested at 80:20, resulting in a hybrid gel with a good consistency for topical application. On day 7 of healing, a punch hole wound in rats, treated with the AgNPs hydrogel had a total wound area reduction of 81.14% compared to 65.11% of hydrogel alone and 45.66% of the control group. Silver-containing hydrogels not only have a great potential to enhance both epidermal and dermal regeneration by accelerating tissue maturation and granulation, but they can also be a powerful tool in the management of infected wounds due to their intrinsic therapeutic properties and the broad-spectrum antimicrobial efficacy (Paladini and Pollini, 2019).

Several studies have also shown that encapsulating therapeutic cells into biomimetic materials is a valuable strategy to protect them from the host’s immune system, providing a chance to reach the tissues and stimulate repair (Kang et al., 2014; Shen et al., 2021; Qin et al., 2022). An efficient method of producing said matrix is to combine decellularised tissue with growth factors or growth-promoting cells, allowing for more efficient tissue regeneration. Hydrogels derived from a-cellular porcine adipose tissue (HAPA) and loaded with adipose-derived stem cells (ADSCs) have been shown to improve skin regeneration, due to biocompatibility and enhanced ability to support cellular growth, differentiation, and ECM formation, which collectively promote effective wound healing and tissue repair (Tan et al., 2019). In addition to being an integral component of the skin, adipocytes have a well-established role in tissue homeostasis and wound healing (Shingyochi et al., 2015; Franz et al., 2018; Kane and Lynch, 2019). Tan and colleagues produced HAPA-ADSC hydrogels and tested them in full-thickness cutaneous excisional wounds. These hydrogels were not only able to induce cutaneous appendages regeneration and angiogenesis in the wound bed, but also increase cell survival when compared to the individual components alone. HAPA was also able to enhance adipogenesis, improving fibroblast migration and epithelialisation thus supporting quality wound closure resulting in fully regenerated skin (Tan et al., 2019).

Techniques that help to maintain cells viability and phenotype within the scaffolds can be used to prolong the longevity of the hydrogel released into the wound bed (Wu et al., 2021). Although the use of stem cells encapsulated into hydrogels has great potential (Qiu et al., 2022), several questions remain as to how to ensure that the cells will differentiate into the desired phenotype once *in vivo*.

Another notable challenge in synthesising the ideal hydrogel is its limitations in vascularisation (Vedadghavami et al., 2017). Angiogenesis is essential to wound healing as it enables the transport of oxygen and nutrients to the wound site, as well as the removal in any waste products produced (Veith et al., 2019). Impaired angiogenesis will result in poor healing or reinfection, while exuberant vessel formation can result in fibrosis and scarring (Qin et al., 2022). Therefore, strategies to improve the bioengineering of hydrogels to achieve optimal angiogenesis are crucial for the successful application of hydrogels for deep wound healing. We have shown that the application of the AnxA1_2-26_ peptide in conjunction with a skin replacement balanced the inflammatory process and modulated angiogenesis by acting as a co-adjuvant regulator of new vessel formation (Lacerda et al., 2018; Fu et al., 2022). Another group demonstrated that a hybrid hydrogel (borosilicate and silk fibroin modified with methacryloyloxy groups) inhibited inflammation and improved angiogenesis via interaction between hypoxia-inducible factor 1-alpha (HIF-1α) and Cu^2+^, thereby promoting wound healing (Pang et al., 2021).

## 5 Injectable *in situ* forming hydrogels

Skin tissue engineering is increasingly using formulations of biomaterials that are easier to handle and address the challenges faced by surgeons, such as filling deep and/or irregular wounds, as reviewed by Halim et al. (2010). Such wounds have poor granulation tissue and would benefit from the engraftment of relevant cell types and growth factors to promote healing. The ideal biomaterial to address this need is a hydrogel that can be mechanically and physiochemically modified for therapeutic intervention to address the clinical need in the management of such wounds.

**FIGURE 5:**
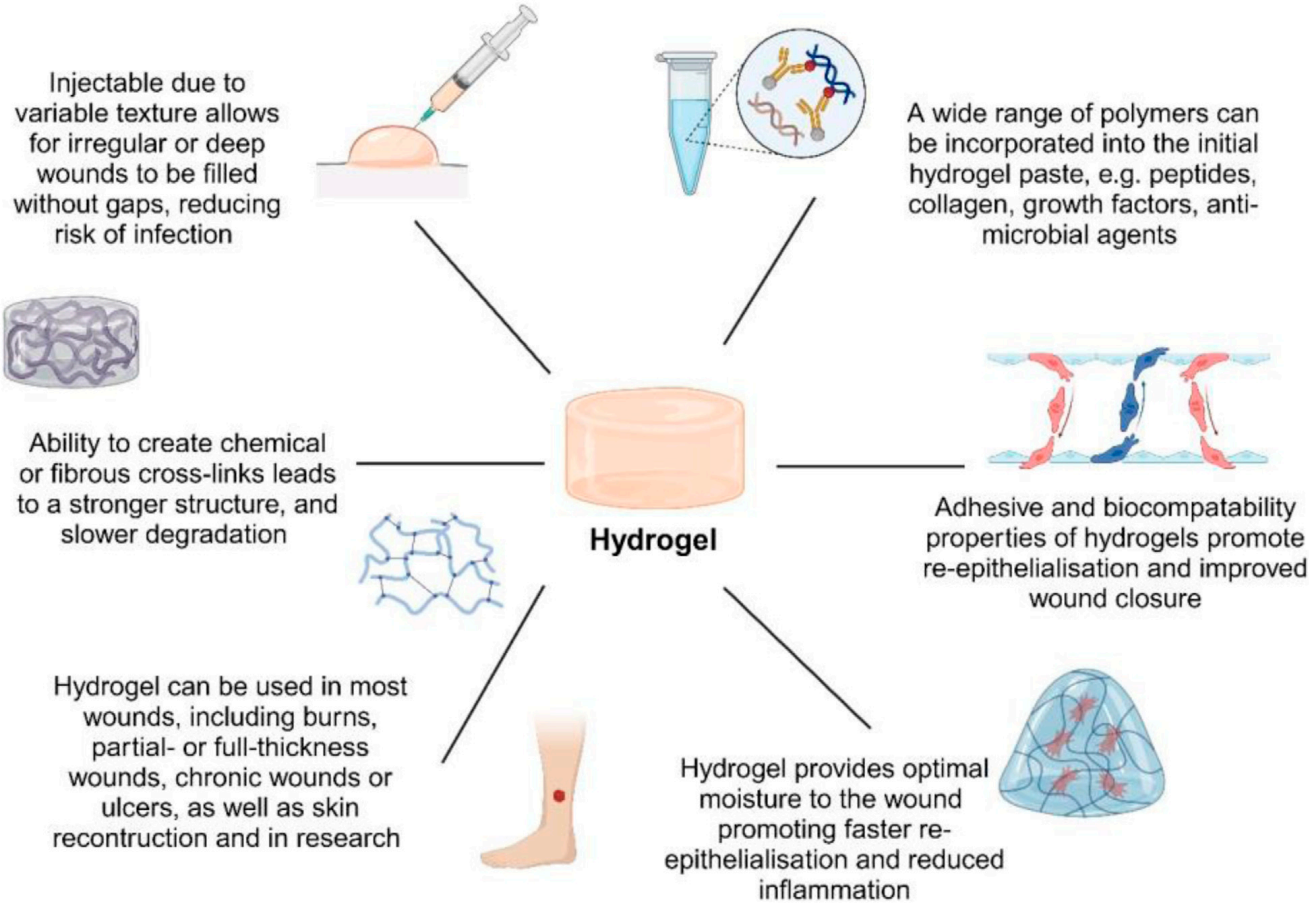
Schematic summary of hydrogel advantages and applications. The enhanced biocompatibility of acellular hydrogels expands their potential for diverse biomedical uses.

Injectable, *in situ* polymerising hydrogels are increasingly being used for biomedical applications such as cell delivery, drug delivery, and/or as scaffolds for reconstruction of injured tissues (Van Vlierberghe et al., 2011). There are many advantages to using an injectable form of hydrogels as this allows access to the target site and deposition of the biomaterial through a minimally invasive approach. One of the challenges of tissue regeneration is the introduction of appropriate cells and biomolecules in a flowable, supportive matrix to the site of injury. In cutaneous wounds, this involves filling complex wound defects, which very often have a multi-tunnel formation and therefore require appropriate formulation and delivery methods (Figure 5).

Hydrogels are well-suited for these applications due to their ability to conform to irregular wound geometries, ensuring uniform coverage and adhesion to the affected area. Additionally, they can be pre-seeded with viable cells (e.g., fibroblasts, mesenchymal stem cells) or incorporated with bioactive molecules (such as epidermal growth factor or vascular endothelial growth factor) without compromising their biological activity during polymerization (Dorsey et al., 2017; Stan et al., 2021; Johnson and Wilgus, 2014). Their degradation rate can be precisely engineered to ensure controlled release of embedded factors. Fast-degrading hydrogels (e.g., gelatin, hyaluronic acid) support acute wound healing, whereas slow-degrading hydrogels (e.g., PEG, PCL) provide long-term structural support for chronic wounds or tissue scaffolding. By tailoring degradation kinetics, hydrogels prevent premature factor depletion and promote timely ECM remodelling, facilitating optimal tissue regeneration (Tsou et al., 2016).

The release of embedded growth factors is usually dependent upon the degradation or destabilisation kinetics engineered into the hydrogel. This can be triggered by physical parameters such as pH change or light-induced scaffold loosening (Park, 1999; Shi et al., 2006; Garbern et al., 2011; Miranda-Calderon et al., 2022). A ready-made flowable hydrogel can be injected to fill wounds and is allowed to polymerise *in situ* after mixing of the ingredients, through chemical or physical cross-linking reactions that occur either during or after injection of the hydrogel precursors (Chen et al., 2017). As with any tissue regeneration scaffold, the mechanical properties of the hydrogel must be close to those of the host tissue to support appropriate differentiation of the seeded or infiltrating cells Cho (Cho et al., 2017).

The ideal hydrogel may therefore be a combination of natural and synthetic polymers, engineered to degrade predictably and functionalised to promote revascularisation, cellular infiltration, and constructive remodelling (Zhu and Marchant, 2011). As with wound dressing hydrogels discussed earlier, the formulation of injectable hydrogels can be tailored to specific functional purposes. Several studies have demonstrated the advantages of using polysaccharides in the creation of injectable hydrogels for wound healing (Huang et al., 2016; Rodell et al., 2016; Dani et al., 2021). Balitaan and colleagues (2020) introduced a novel type of hybrid composite hydrogel in their research. This hydrogel was created by combining acrylamide-modified β-chitin with alginate dialdehyde using a popular process called known as cross-linking. The resulting hydrogel exhibited remarkable properties such as biocompatibility, biodegradability, and injectability. The authors examined the self-healing capacity of the hydrogels by both macroscopic observations and rheological investigations, which is quite intriguing. When zebrafish were used as a model, the wounds that were treated with the suggested hydrogels showed around 87% healing of the wound area, but untreated wounds only had about 50% closure (Balitaan et al., 2020).

Lokhande et al. suggested using composite carrageenan-based gels containing synthetic 2D nanosilicates to improve the process of wound healing. Introducing 2D nano-silicates led to an increase in protein adsorption, which in turn improved cell adhesion and spreading. Additionally, it promoted greater binding of platelets and shortened the time it takes for blood to clot (Lokhande et al., 2018). Hu et al. (2020) developed an injectable hydrogel that can release drugs specifically during the inflammatory stage. This hydrogel is responsive to changes in pH and reactive oxygen species (ROS), and it also has the ability to self-heal and reshape itself. The researchers attached phenylboronic acid to the side chain of the alginate polymer. This modification resulted in the polymer having antibacterial and anti-inflammatory capabilities. They achieved this by enclosing the antibiotic amikacin and the anti-inflammatory medication naproxen in micelles (Huang et al., 2016).

Scientists have also examined the use of antioxidant and anti-inflammatory substances discovered in fruits to modify hydrogels for the purpose of mending wounds. Zhang W. et al. (2020) developed injectable hydrogels using carboxymethyl chitosan and oxidised hyaluronic acid that contained blueberry anthocyanins (BA), known for their antioxidant and anti-inflammatory effects, in a dual hybrid-composite hydrogel (Zhang W. et al., 2020). The findings indicated that the gelation time and mechanical characteristics of oxidised hyaluronic acid were enhanced. The hydrogel greatly expedited the wound healing process in a rat model with full-thickness skin wounds by stimulating the growth of new skin cells and tissues, reducing inflammation, and facilitating the production of collagen and the formation of new blood vessels. Furthermore, the hydrogel resulted in an increased expression of VEGF and IL-10 proteins, while the levels of NF-κB were decreased. Additionally, it facilitated the conversion of macrophages from the M1 to the M2 phenotype. The findings indicated that the injectable hydrogel utilised in this investigation exhibited synergistic properties in enhancing the process of wound healing (Qin et al., 2022).

Diabetic wounds pose a significant challenge because they are often infected with bacteria that are resistant to several drugs, and they occur in a wound microenvironment that is characterised by high blood sugar levels and oxidative stress. In order to address these challenges, Wang and his colleagues suggested the use of an injectable hydrogel that possesses multiple functions. This hydrogel is created through a Schiff-based reaction between ε-polylysine-coated MnO2 nanosheets and insulin-loaded self-assembled aldehyde Pluronic F127 micelles. The hydrogel has demonstrated exceptional antimicrobial abilities against bacteria that are resistant to multiple drugs. The MnO2 nanoenzyme catalyses the decomposition of endogenous H_2_O_2_ into H_2_O and molecular O2, effectively controlling the harmful oxidative conditions in a wound microenvironment. Furthermore, the hydrogel, which is susceptible to changes in pH and redox conditions, exhibited a regulated release of insulin that was sustained over time and could be managed in terms of both space and time (Matai et al., 2020).

Bai and his colleagues have suggested a self-healing hydrogel for treating diabetic foot ulcers. This hydrogel is created in the affected area by combining N-carboxyethyl chitosan and adipic acid dihydrazide with hyaluronic acid-aldehyde by crosslinking. The hydrogel created a damp and anti-inflammatory setting that stimulated the release of growth factors from bone marrow mesenchymal stem cells, hence expediting the process of wound healing (Bai et al., 2020).

Although there is current promising data in the field of injectable hydrogels for wound healing, current challenges in the delivery and gelation system of the hydrogels.

To achieve *in situ* gelation, hydrogels for skin applications require significant gelation kinetics under mild conditions (close to physiological pH) and an LCST below human body temperature in order to form a stable topical hydrogel (Chatterjee et al., 2018). Various techniques have been discussed to safely deliver the hydrogel into the wound site, allowing for consistency, preservation, retention and viability of the biomolecules and cells at the injection site (Chen et al., 2017). However, rheology, viscosity, storage, and injection force of the hydrogel need to be well evaluated and tuned for a successful translation into clinics.

To overcome the limitations of the *in situ*-forming hydrogels, they have been engineered with supramolecular chemistry, such as shear-thinning, which is the ability of a material to decrease in viscosity with increasing shear. Such hydrogels can be loaded (pre-formed) into syringes, extruded upon application of shear, and rapidly re-formed when the load is removed, a process also known as self-healing (Chen et al., 2017).

The self-healing nature of these biomaterials has notable advantages, such as allowing material retention at the injection site (wound bed), and offering *in situ* polymerisation, minimising the risk of embolization Nevertheless, they may not provide the mechanical stability of the traditional *in situ* cross-linking covalent systems, often requiring secondary cross-linking techniques to enable stabilisation after injection (Rodell et al., 2016; Wang et al., 2017). In particular, dynamic covalent cross-links have been explored, whereby changes in the dynamic nature confer shear-thinning during injection and then slow self-healing after injection (Huang et al., 2016). For a successful translation, the precise injectability of the hydrogels is essential to enable minimally invasive delivery, which can be achieved through various methods, such as percutaneous catheters or via minimally invasive or robotic surgical techniques (Rodell et al., 2016; Chen et al., 2017). For a homogeneous and reproducible mixing of the hydrogel components, a “two-way” delivery method has been proposed, whereby two syringes are connected, one filled with a fluid suspension of cells (and/or carrying bioactive factors, biomolecules, drugs) and the other with a viscous gel containing the photoinitiator, crosslinker or other chemicals (Dani et al., 2021). And this is a very promising tool that can be used in wide range of clinical applications, from wound healing and tissue regeneration to drug delivery and cancer therapy.

## 6 Commercially available hydrogels and injectable hydrogels

Currently, the vast majority of commercially available hydrogels for wound healing act as wound dressings in the form of topical gel products (DermaSyn, from DermaRite; Purilon®gel, from Coloplast) hydrogel sheets (Neoheal^®^ Hydrogel, from Kikgel; Simpurity™ Hydrogel, from Safe n’ Simple) or hydrogel soaked films or gauzes (DermaGauze™, from DermaRite; Restore Hydrogel, from Hollister Incorporated) (Aswathy et al., 2020; Firlar et al., 2022; Gounden and Singh, 2024).

Topical gel products are available in a semi-liquid form that can be applied directly into the wound. Their formulation allows for frequent reapplication that can be adapted to the exudate secretion rate from the wound. It is often recommended for burns or ulcers. In a similar way, hydrogel-soaked gauzes are able to absorb exudate and can be easily removed and reapplied depending on the exudate present in the wound. However, they are able to isolate the wound providing a moisture-rich environment for wound healing. Hence, they are more versatile for different types of wounds. On the other hand, hydrogel sheets provide a longer-lasting moisture environment but with low exudate absorption rate. Hydrogel sheets are therefore suited for more superficial wounds and minor burns with low exudate level (Aswathy et al., 2020; Firlar et al., 2022; Gounden and Singh, 2024). Although single-polymer dressings are available (HELIX3-CM^®^ Collagen Matrix, AMERX Healthcare), most commercially available formulations consist of hybrid or composite hydrogels. Interestingly, although current research leans towards natural polymers or biomimetic hydrogels, there is a clear commercial preference in the use of synthetic or organic-inorganic hybrid hydrogels, potentially due to them being easily scalable for industrialisation (Aswathy et al., 2020; Firlar et al., 2022; Gounden and Singh, 2024).

Besides the wide range of products available as wound dressing, there is a gap in the market regarding injectable hydrogels for skin wound healing. Most FDA-approved injectable hydrogels for biomedical applications are restricted to cosmetic surgery, with some being used for cancer therapy (Mandal et al., 2020; Almawash et al., 2022; Zhao et al., 2024). There is also a number of injectable hydrogels under clinical trials for various therapeutical purposes, including cancers, incontinence or osteoarthritis (Mandal et al., 2020). No clinical trials within the scope of injectable hydrogels for skin wound healing treatment are active, as reported in clinicaltrials.gov (last accessed September 2024).

There are several challenges that contribute to the lack of commercially available injectable hydrogels for wound healing, even though there is stablished evidence that support the benefits in *in vitro* and *in vivo* settings. From a technical point of view, there is generalised low chemical and physical reproducibility in hydrogel formation, which translates to high batch-to-batch variability. There are also mechanical concerns within hydrogels’ long-term structure, particularly when it has been modified to support injectability (Øvrebø et al., 2022). Moreover, the polymers used are all subject to regulatory approval, which can become an issue when relying on synthetic polymers. In addition, as discussed earlier, novel research has shown the benefits of hybrid-composite multifunctional hydrogels (Wang X. et al., 2024). Multifunctional hydrogels do not only need to support cell growth and proliferation and ensure complete degradation at a pre-defined rate, but a lot of newly developed scaffolds are also used as drug/bioactive compound delivery systems. As drug-delivery systems, hydrogels become a combination product from a regulatory perspective and require a longer approval process (up to 12 years) (Alonso et al., 2021; Almawash et al., 2022).

## 7 Future perspectives

The foundational properties of hydrogels themselves has propelled their reputation as a durable and adaptable biomaterial for all aspects of wound healing, creating a wide prospect for further research into their applications. Hydrogels have become one of the most promising and evolving therapies in wound management due to their versatility, including functionalisation with biomolecules such as antimicrobial compounds, immunomodulatory molecules, and growth factors, or being loaded with growth-promoting cells. They can also be physiochemically modified to mimic the natural microenvironment, thereby imparting additional regenerative and healing properties (Qin et al., 2022).

This functionality opens up the possibility of using the hydrogels in a minimally invasive way and for a range of uses, some of which including achieve the healing of chronic deep wounds, and to be used as a bio-ink for 3D printing to create a personalised template unique to the host’s wound, either pre-loaded with cells (Matai et al., 2020), or electrospun to create a more complex structure (Gao et al., 2021). Recent studies have described the most advanced approach of Four-dimensional (4D) -printing to be increasingly favourable and advantageous when compared to 3D printing, as it provides a scaffold with the ability to morph its shape over short durations. This results in improved biocompatibility and the potential for increased clinical applications in complex cases (Ramezani and Mohd Ripin, 2023).

4D printing, an advancement of 3D printing, incorporates time-dependent transformations to produce materials that respond to environmental alterations, providing novel solutions in wound healing. Injectable hydrogels, composed of biocompatible polymers such as alginate or hyaluronic acid, replicate the extracellular matrix and offer a conducive environment for tissue regeneration (Yarali et al., 2024). Augmented by 4D printing, these hydrogels dynamically respond to wound-specific stimuli, either hardening for support or relaxing to facilitate cellular penetration. They can administer pharmaceuticals or stem cells in a regulated manner and possess the capability for self-repair (Lu et al., 2024). The process in general has proved to be an appealing alternative to conventional production methods due to its demonstrated ability to design the scaffold with its desired behaviours, and fabricate real-time adaptive structures using a wide range of components and biomaterials, and in a timely manner (Appuhamillage et al., 2024). Despite obstacles in scalability and control, these versatile hydrogels signify revolutionary progress in customised wound treatment and provide further scope for use in biomedical engineering and soft robotic.

## 8 Conclusion

The use of hydrogels in recent years has increased exponentially, due to recognition of their wide range of properties and applications. Although the predominate features associated with hydrogels relate to their biocompatibility and clinical uses, studies have demonstrated an array of additional lesser-known advantages. Hydrogels are initially composed of a 3-dimentional fibrous scaffold and extracellular matrix to provide a suitable environment for growth of surrounding tissue. However, as research into hydrogels advanced it would found that the addition of growth promoting biomolecules and polymers into the extracellular matrix or seeded onto the scaffold would optimise the environment for growth promotion and increase cellular integration in the host. With this in consideration, most commercially available hydrogels incorporate a range of biological molecules, including proteins present in native skin such as collagen and elastin, and polysaccharides such as hyaluronic acid and sugars. The addition of biomolecules results in improves biocompatibility, non-toxic biogradability, and mechanical strength.

Over the last decade, particular attention has been shown in relation to hydrogel production methods, due to their increase in demand, and wide scope for potential applications. Conventional methods include chemical and physical crosslinking, and the combination of amphiphilic grafts and block polymers which consists of both hydrophobic and hydrophilic molecules with the ability to self-assemble into a scaffold once combined. Recent studies have investigated the rise of 3D- and 4D printing to produce hydrogels, as these approaches have sparked interest within the tissue engineering field, and soft robotics. The main appeal of multi-dimensional printing surrounds the ability to produce a hydrogel with programmed behaviours, capable of changing its properties in response to external stimuli, such as temperature, light, pH and humidity (Ijaz et al., 2023). As research into these biomaterials has progressed and advancements have been made, recent literature demonstrates that hydrogels within the current market are being used widely within clinical settings, particularly for would care due to their excellent biocompatibility, high water content, shape morphology, and swelling ratios, as well as a scaffold to implement drug delivery systems. Despite major advances in recent years in the development of new methods to synthesise hydrogels in accordance with their desired use, the complexity of both natural ECM-derived and synthetic hydrogels is still poorly understood. As previously outlined, the versatility and adaptability of hydrogels is continuously being researched and so the scope of what can be achieved through the use of hydrogels is growing substantially. Therefore, deepening our knowledge on the biomaterial behaviour will have a tremendous impact on the control of constructs, providing a degree of engineerability to the ECM-derived hydrogels and a more tailored immunomodulatory and pro-regenerative properties to the synthetic/hybrid ones (Mora-Solano and Collier, 2014; Hotaling et al., 2015; Anna-Lisa et al., 2024). Hydrogels hold immense potential for future developments and applications into more niche areas of medicine, such as treating fractures by improving bone regeneration. The main limitations of hydrogels, including their thermos-photosensitivity and potential non-biogradability, will be areas of initial investigation in order to achieve an enhanced hydrogel with a longer shelf-life and improved biocompatibility. With all aspects of these biomaterials considered, future developments within the would healing field will prove increasingly necessary as the demand for hydrogels, along with their popularity, are both growing exponentially.

The intrinsic versatility, biocompatibility, and adaptability of hydrogels have positioned them as a key biomaterial in wound healing, tissue engineering, and regenerative medicine, offering a vast scope for further advancements. Their ability to be functionalized with bioactive molecules—such as antimicrobial agents, growth factors, and immunomodulators—or pre-seeded with therapeutic cells enhances their regenerative efficacy. Furthermore, their tunable physicochemical properties allow precise modifications to mimic the extracellular matrix (ECM), providing structural and biochemical support for tissue repair and cellular integration (Qin et al., 2022).

Recent innovations in hydrogel engineering have enabled the development of biomimetic designs, incorporating key ECM components such as collagen, elastin, and hyaluronic acid. These advancements not only improve mechanical stability and degradation control but also enhance cell adhesion, migration, and proliferation, optimizing hydrogel performance for wound care, drug delivery, and scaffold-based tissue regeneration. Additionally, emerging fabrication techniques, including 3D and 4D printing, have allowed the creation of stimuli-responsive hydrogels capable of adapting to environmental cues such as temperature, pH, light, and humidity, expanding their applications into tissue engineering, smart biomaterials, and soft robotics (Ijaz et al., 2023).

Despite significant progress, challenges remain in optimizing hydrogel properties to fully harness their potential. The complexity of both natural ECM-derived and synthetic hydrogels requires further exploration to improve their mechanical robustness, degradation kinetics, and immunomodulatory capabilities. A deeper understanding of hydrogel behavior will allow for greater control over engineered constructs, providing tailored regenerative and therapeutic functionalities (Mora-Solano and Collier, 2014; Hotaling et al., 2015; Anna-Lisa et al., 2024).

Future research should focus on addressing key limitations, such as thermo- and photosensitivity, biodegradability, and long-term stability, which will be essential for enhancing hydrogel longevity and efficacy in clinical settings. Expanding hydrogel applications beyond wound healing, into areas such as bone regeneration, neural tissue engineering, and organ repair, highlights their transformative potential in medicine. With the growing clinical demand and rapid advancements in biomaterial science, hydrogels hold immense potential to redefine regenerative medicine, ensuring more effective, personalized, and long-lasting therapeutic solutions. Continued interdisciplinary research will be fundamental to unlocking new frontiers in biomedical innovation, paving the way for next-generation hydrogels with enhanced functionality, responsiveness, and translational success.
